# Role of the Ionomer in Supporting Electrolyte-Fed Anion Exchange Membrane Water Electrolyzers

**DOI:** 10.1021/acselectrochem.4c00061

**Published:** 2024-11-06

**Authors:** Emily K. Volk, Arielle L. Clauser, Melissa E. Kreider, Diego D. Soetrisno, Sunilkumar Khandavalli, Joshua D. Sugar, Stephanie Kwon, Shaun M. Alia

**Affiliations:** † Advanced Energy Systems Graduate Program, Colorado School of Mines, Golden, Colorado 80401, United States; ‡ Sandia National Laboratories, Livermore, California 94550, United States; § Chemistry and Nanoscience Center, National Renewable Energy Laboratory, Golden, Colorado 80401, United States; ∥ Materials Science Center, National Renewable Energy Laboratory, Golden, Colorado 80401, United States; ⊥ Department of Chemical and Biological Engineering, Colorado School of Mines, Golden, Colorado 80401, United States

**Keywords:** green H_2_, anion exchange membrane electrolyzer, catalyst layer, electrolysis, ionomer

## Abstract

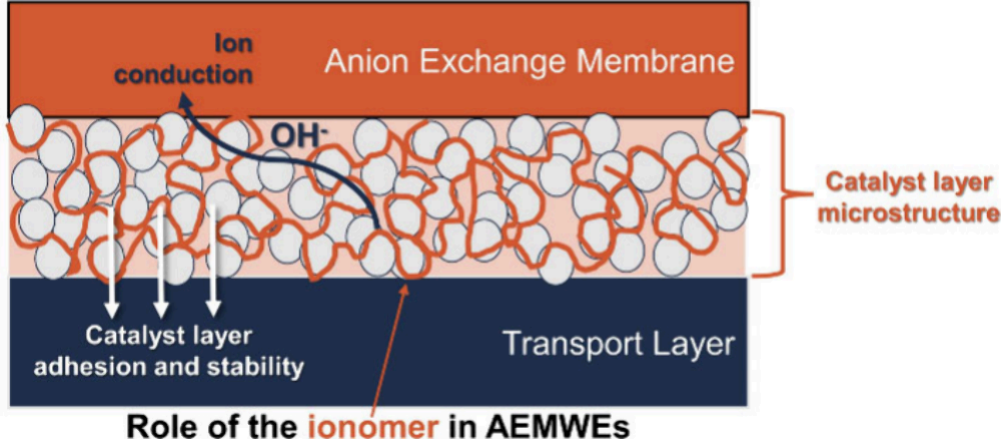

While anion exchange membrane water electrolyzers (AEMWEs) have achieved significant performance advances in recent decades, overpotentials remain high relative to their proton exchange membrane water electrolyzer (PEMWE) counterparts, requiring AEMWE-specific catalyst layer design strategies to further advance this technology. In this work, the role of the ionomer in catalyst layer structure and quality, catalyst layer stability, and ion conduction for supporting electrolyte-fed AEMWEs is assessed for catalyst layers composed of NiFe_2_O_4_ and PiperION TP85 from Versogen at variable ionomer contents (0–30 wt %) for tests up to 200 h. The results reveal that, for supporting electrolyte-fed AEM devices, the ionomer is not required for ion conduction through the catalyst layer. Instead, the ionomer is found to play a critical role in catalyst layer structure and stability, where intermediate ionomer contents lead to the lowest overpotentials, highest effective surface areas, and lowest catalyst layer resistances. Catalyst layer stability is found to be a function of both catalyst adhesion and ionomer loss. These results show that an ionomer may be selected which is not of the same chemistry as the anion exchange membrane, mitigating ionomer stability concerns throughout the catalyst layer and offering a pathway towards highly active and stable AEMWEs.

## Introduction

**Figure 1:**
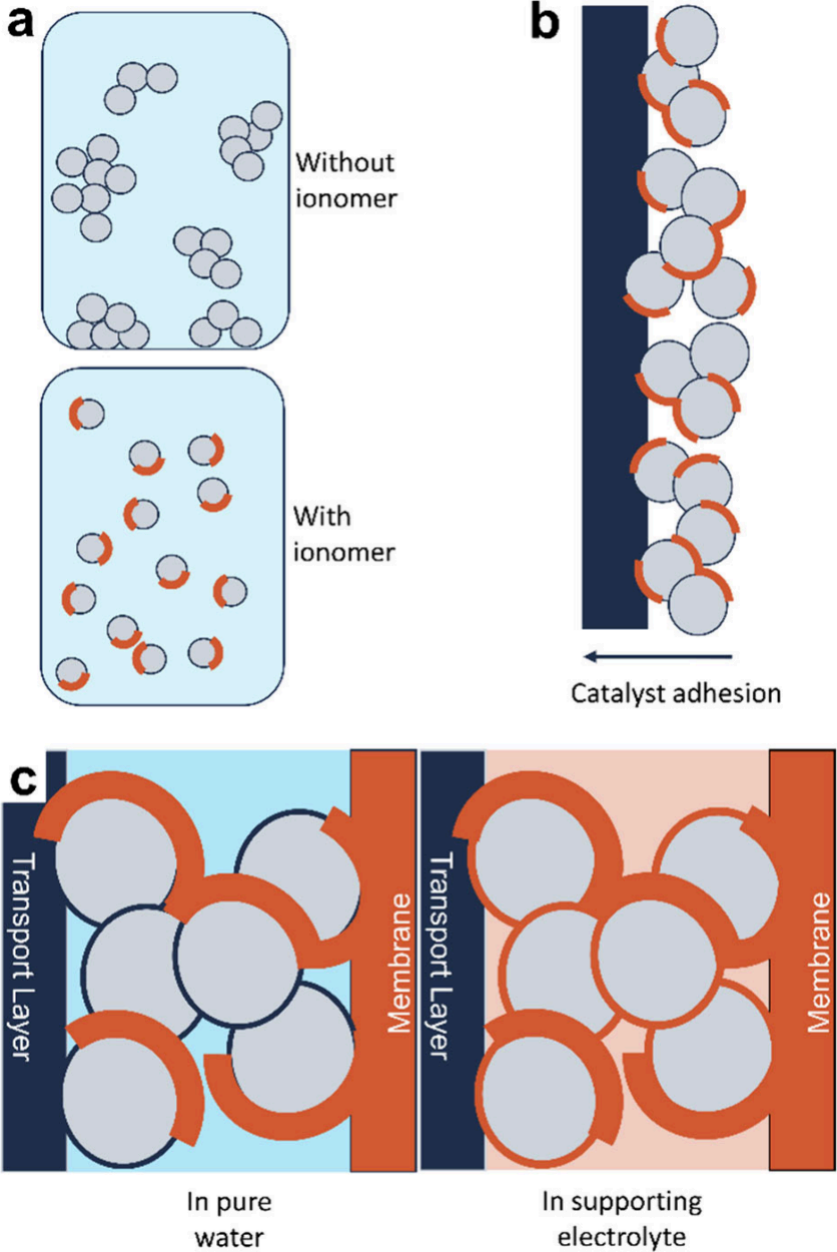
Proposed roles of the ionomer in AEMWEs, including (a) in dispersing the agglomeration structure of catalyst particles, (b) providing adhesion and binding of catalyst particles to a substrate (membrane or transport layer), and (c) in providing ionic transport pathways. In all parts, areas shown in orange represent regions and surfaces accessible to ionic transport. In (c), the pure water case, ionic transport is only accessible in regions where the ionomer is in a continuous network. In the supporting electrolyte case, all regions and surfaces that are in contact with ionomer and/or supporting electrolyte are accessible to ion transport.

Ion exchange polymers, or ionomers, are frequently employed in the catalyst layers of low temperature electrochemical devices, including fuel cells, water electrolyzers, and CO_2_ electrolyzers. There are several proposed functions for the ionomer (Figure 1). First, the ionomer can affect the microstructure of catalyst inks, which can subsequently affect catalyst layer structure (Figure 1a), as previously shown for proton exchange membrane fuel cells (PEMFCs)^1^ and water electrolyzers (PEMWEs).^2^ Similar effects have been proposed for anion exchange membrane water electrolyzers (AEMWEs),^3,4^ although catalyst layer structures with and without an ionomer have not yet been demonstrated in literature. Second, the ionomer can bind catalyst particles to the electrode substrate (i.e., the membrane or transport layer), providing sufficient adhesion to prevent catalyst detachment or delamination (Figure 1b). Lastly, the ionomer can serve as a transport network for charged ions through the catalyst layer and between the catalyst layer and the membrane (Figure 1c). For PEM systems, which are operated in pure water, an ionomer is required to transport H^+^ ions between catalytic active sites and the proton exchange membrane. AEMWEs also commonly utilize an ion exchange membrane and corresponding ionomer to provide transport for ions through the cell. AEMWEs, however, are often operated with supporting electrolyte (e.g., 0.1–1 M KOH, KHCO_3_, or K_2_CO_3_) due to significant performance (activity and stability) advantages over pure-water operation.^5−7^ As supporting electrolyte provides its own ion conductivity, an ionomer may not be required to provide ion conductivity within the catalyst layers, posing questions regarding the importance and specific roles of ionomers in AEMWE systems.

Recent efforts have led to significant advances in the development of anion exchange polymers, including several commercially available materials.^8^ These efforts have largely focused on improving membrane properties, including balancing ion exchange capacity with water uptake and swelling concerns, and designing for chemical stability.^9−11^ Conventionally, the same type of polymer is often used for both membranes and ionomers without further consideration, even though the specific properties required for ionomers may be different from those required for membranes. For example, anion exchange ionomers have been shown to react and degrade at highly oxidative conditions of AEMWEs (high pH, high potential), leading to catalyst layer instability and cell losses.^6,7,12−16^ Lindquist et al. investigated different routes of ionomer failure in AEMWEs with IrO_2_ anode catalysts, and found that ionomer degradation caused by oxidative instability was the predominant mechanism of cell failure for three different ionomer types in pure water feeds.^7^ Li et al. demonstrated that phenyl groups in ionomers strongly adsorb to catalytic active sites, blocking these sites from performing the oxygen evolution reaction (OER) and leading to preferential oxidation and degradation of the ionomer, and subsequent cell performance losses over time.^15^ Such polymer degradation routes are expected to be most critical at the anode, where potentials are high. Koch et al. utilized single-cell testing with post-mortem transmission electron microscopy (TEM) to show that ionomer films encompassed catalyst particles when ionomer content was higher than 15 wt %.^17^ The electronically insulated catalyst particles then showed decreased accessible active surface areas, thus leading to lower cell activities.

Most previous research in AEMWE has focused on optimizing ionomer content in the anode catalyst layer with a consensus that intermediate ionomer content (5-20 wt %) lead to higher cell activities.^3,4,17^ These works proposed that intermediate ionomer content balances the issues of catalytic site blocking (high ionomer content) and mechanical instability (low ionomer content), although all of these tests were only verified during short-term testing (< 24 h). While these works demonstrated the importance of optimizing ionomer content for higher cell performance, the specific roles for the ionomer in AEMWEs have remained unvalidated. Furthermore, the effect of ionomer type and content on long-term cell operation is yet unknown.

In this work, Versogen PiperION TP85 ionomer content was systematically varied from 0 to 30 wt % for anode AEMWE catalyst layers with NiFe_2_O_4_ catalysts to understand the impacts of ionomer content on overall cell losses. PiperION TP85 was chosen for its commercial availability and NiFe_2_O_4_ has been identified as a candidate for a commercial benchmark catalyst for alkaline OER.^5,18^ The results show that a high ionomer content leads to decreased active site accessibility and heterogeneity in the catalyst layer, as demonstrated by electrochemical and top-down and cross-sectional scanning electron microscopy (SEM) with energy dispersive spectroscopy (EDS). Durability testing (200 h) results show that cell losses are caused by catalyst detachment facilitated by ionomer loss, evidenced by ex-situ X-ray diffraction (XRD), X-ray photoelectron spectroscopy (XPS), and inductively coupled plasma mass spectrometry (ICP-MS) results. A non-anion conducting polymer, K^+^-exchanged Nafion, achieved cell activities which exceeded those with the Versogen ionomer, indicating that ion conduction by the ionomer is not required when operating in supporting electrolyte. As such, there is no need for the chemistry of the ionomer phase to necessarily match the chemistry of the anion exchange membrane for supporting electrolyte-fed AEMWEs. These results suggest that the future ionomer design should focus on chemical stability, binding quality, and the ability to facilitate homogeneous and porous catalyst layers.

## Materials and Methods

### Catalyst Materials

All catalytic materials were obtained from commercial suppliers and used without further treatment. NiFe_2_O_4_ (US Research Nanomaterials, 30 nm, 98%) was used for all anode catalyst layers and Pt/C (47% Pt on C, Tanaka Kikinzoku Kogyo, TEC10E50E) was used for all cathode catalyst layers.

### Physical and Electrochemical Characterization of Catalyst Layers

The double layer capacitance was determined from cyclic voltammograms (CVs) at three scan rates (100, 50, and 20 mV/s). Dynamic light scattering (DLS) experiments were conducted at 25°C using a Zetasizer Nano ZS (Malvern Instruments Ltd, Malvern, U.K.) with at least five readings to ensure repeatability of the measurements. Inks for DLS measurements contained 0.05 wt % solids and a solvent and ionomer content that matched those for MEA testing. Settling experiments were conducted on inks of the same composition as those for MEA testing (see below) using a custom-built, closed chamber which contained light emitting diode (LED) light strips and a camera (Logitech C615), and images were collected at 2-h intervals for 10 days. An FEI Helios NanoLab 600i DualBeam scanning electron microscopy/focused ion beam (SEM/FIB) was used to collect top-down images using both a solid-state circular backscatter (CBS) detector at 5 kV and 0.2 nA and Everhart-Thornley detector (ETD) to collect secondary electron images. Energy dispersive X-ray spectroscopy (EDS) maps were collected at 15 kV and 6.4 nA using Oxford’s AZtec software and Oxford 170 mm^2^ Ultim Max SDD EDS detector. Focused ion beam cross sections were made in representative areas of the samples and corresponding EDS maps were collected with the same parameters as top-down images. Additional top-down images were collected with a Hitachi S-4800 SEM with Thermo-Fisher EDS detector at 15 kV and 10 μA. Before imaging, the ionomer in the catalyst layers was ion exchanged from CO_3_^2–^ or OH^–^ to I^–^ for improved visualization of the ionomer phase (details in Section S1 of the Supporting Information (SI)). Anode and cathode catalyst loadings were measured (3× 30s exposures) via X-ray fluorescence (Fischer XDV-SDD XRF). X-ray diffractograms (XRD) were obtained (2θ = 13.5° – 88°) using a Bruker D8 Discover with Cu K-α radiation (λ = 1.5406) and general area detector diffraction system (GADDS). X-ray photoelectron spectroscopy (XPS) characterization of C, N, O, and F in tested catalyst layers was conducted at the Stanford Synchrotron Radiation Lightsource (SSRL) at beamline 8-2 using a photon energy of 960 eV, pass energy of 200 eV, and energy steps of 0.05–0.1 eV. The samples were neutralized using an electron flood gun. The spectra were calibrated by shifting the C–C 1s peak to 284.8 eV. The spectra were analyzed and fit using CasaXPS software^19^ as has been reported previously.^20^ Inductively coupled plasma-mass spectrometry (ICP-MS) measurements were conducted using a Thermo Scientific iCAP Q instrument in kinetic energy discrimination mode using He cell gas. Aliquot samples taken from the electrolyte reservoir were diluted 20× with 2% HNO_3_ (Fischer Chemical, Optima Grade, 67–70%), to achieve a final concentration of 0.05 M KOH.

### Electrode Construction

Anode catalyst layers were sprayed onto Ni mesh (Bekaert BEKIPOR 2NI 19-0.25) porous transport layers (mounted to a vacuum hot plate, 80 °C) using a hand airbrush. Anode ink dispersions were formulated to target 0.5 mg cm^–2^ of catalyst (metal) and varying ionomer contents (0–30 wt %) and consisted of 10 vol % deionized water (DIW) and 90 vol % *n*-propanol with a solids content of 2 wt %. PiperION TP85 from Versogen (5 wt % dispersion in Ethanol) and Nafion perfluorinated resin solution (5 wt % dispersion in water and alcohols, Sigma-Aldrich, 527084) were used as ionomers. In this work, ionomer content (reported in wt %) refers to the percentage of the total solids mass that is composed by the ionomer phase. Cathode catalyst layers were directly sprayed onto Versogen PiperION membranes (TP-85, 80 μm) using a Sonotek spray station with Accumist nozzle. Cathode ink solutions were formulated to target 0.3 mg cm^–2^ of Pt and 30 wt % Versogen PiperION-A TP-85 ionomer solution (5 wt % in ethanol) and consisted of 57 vol % DIW and 43 vol % *n*-propanol. Before preparing the electrodes, the Nafion was ion-exchanged from H^+^ to K^+^ form to prevent possible dissolution of catalyst materials during spraying procedures induced by H^+^ acidic sites; the details of this procedure are discussed in Section S2 of the SI.

### Membrane Electrode Assemblies (MEAs)

All tests were performed using single-cell MEAs with a 5 cm^2^ active area. MEAs consisted of a carbon paper gas diffusion layer (Fuel Cell Earth, MGL280, 80280-40) on the cathode side, a catalyst-coated Ni mesh porous transport layer on the anode side, and Versogen 80 μm membranes with the Pt/C catalyst coated on the cathode-facing side. All MEAs were tested in cell hardware consisting of Al endplates (Fuel Cell Technologies), Au coated current collectors, and Ni triple serpentine flow fields. Membranes were pretreated via soaking in 3 M KOH for 48 h (refreshed once at 24 h) and the catalyst-coated Ni mesh transport layers were pretreated via soaking in 3 M KOH for 24 h before use.

### Electrochemical Testing

All experiments were performed using a two-electrode configuration and reported potentials are cell potentials. Cell overpotentials were calculated based on the thermodynamic potential (1.178 V), which was determined for the non-standard temperature and pressure conditions as detailed in Section S3 of the SI. Electrochemical measurements (including polarization curves, cyclic voltammetry, and electrochemical impedance spectroscopy) were taken using an Autolab PGSTAT302N potentiostat (Eco Chemie, Metrohm Autolab). 200-h durability tests were conducted via constant potential holds at 1.8 V, with polarization curves taken every 2 h. Electrochemical activities were determined from potentiostatic polarization curves (120 s per voltage) collected at 1.40, 1.42, 1.44, 1.46, 1.48, 1.50, 1.55, 1.60, 1.65, 1.70, 1.80, 1.90, and 2.0 V. Electrochemical impedance spectroscopy was collected at the same potentials as the polarization curves as well as three non-Faradaic voltages (1.20, 1.25, and 1.30 V) for catalyst layer resistance (*R*_CL_) calculations, as has been published previously.^18,21^

## Results and Discussion

### The Role of the Ionomer in Anode Catalyst Layer Structure and Active Site Accessibility

**Figure 2:**
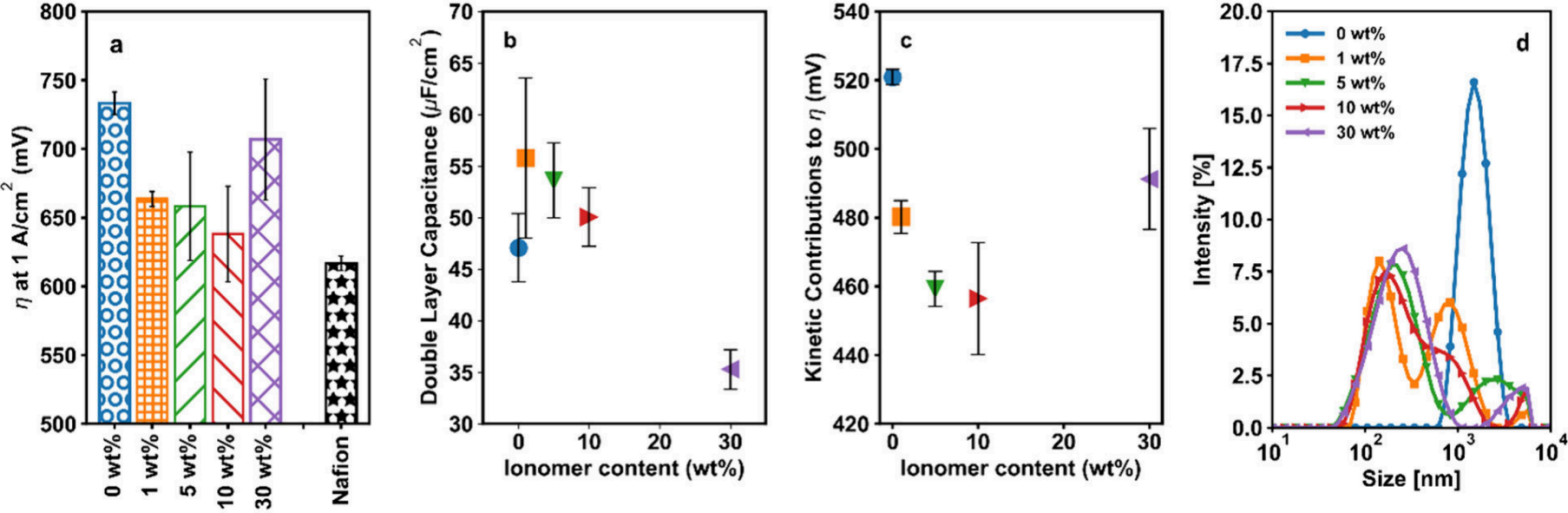
Results from tested membrane electrodes assemblies with different ionomer contents including (a) cell overpotentials at 1.0 A-cm^–2^, (b) the double layer capacitance calculated from cyclic voltammetry, (c) and kinetic overpotentials. All electrochemical results shown are the average and standard deviation of three repeated runs. (d) Intensity distribution profile for the particle sizes of NiFe_2_O_4_ dispersions at different Versogen ionomer contents.

Figure 2a shows the changes to overpotentials at 1 A-cm^–2^ as the ionomer content was varied between 0–30 wt %. A volcano-like relationship was observed for cell activities and ionomer content, where the samples with intermediate ionomer content had the lowest overpotentials (highest activities), and the samples with very low and very high ionomer content had the highest overpotentials (lowest activities). Specifically, the sample with no ionomer had the highest overpotential (733 ± 8 mV), which decreased significantly (to 664 ± 5 mV) when only a small amount of ionomer (1 wt %) was incorporated. Increasing the ionomer content further resulted in a decrease in overpotential; the samples with 5 and 10 wt % ionomer had overpotentials of 658 ± 39 and 638 ± 35 mV, respectively, nearly 100 mV lower than the 0 wt % sample. Further increasing the ionomer content, however, resulted in increased overpotentials, as shown for the sample with 30 wt % ionomer (707 ± 44 mV), partially due to the blocking of active sites by the ionomer phase, as discussed next.

Double layer capacitance (*C*_DL_; Figure 2b and Table 1) measurements were employed to study the effect of ionomer content on active site accessibility. The *C*_DL_ is considered to be a proxy for the electrochemically accessible surface area for non-PGM oxide materials,^18,22,23^ and provides a reliable comparison among samples with the same chemical identity.^23^ We note that the results here were calculated from cell potentials (not half-cell potentials), and thus the CVs may include features from both the anode and cathode catalyst layers and transport layers; the measured trends in *C*_DL_, however, mainly reflect the trends from the anode catalyst layers as only the ionomer content in the anode catalyst layer was varied for each experiment.

**Table 1:** Values for the Overpotential at 1 A/cm^2^, Kinetic Contributions to Overpotential at 1 A/cm^2^, *R*_CL_, HFR, and Tafel Slope^a^

sample	η at 1 A/cm^2^ (mV)	kinetic η (mV)	*R*_CL_ (Ω)	HFR (Ω)	Tafel slope (mV/dec)
0 wt % Versogen	733 ± 8	521 ± 2	502 ± 42	86 ± 6	105 ± 1
1 wt % Versogen	664 ± 5	480 ± 5	467 ± 29	83 ± 1	97 ± 6
5 wt % Versogen	658 ± 39	459 ± 5	341 ± 45	93 ± 9	90 ± 5
10 wt % Versogen	638 ± 35	456 ± 16	357 ± 70	88 ± 2	85 ± 4
30 wt % Versogen	707 ± 44	491 ± 15	771 ± 13	86 ± 7	99 ± 5
10 wt % Nafion	617 ± 5	460 ± 6	450 ± 5	84 ± 2	90 ± 3

a Values presented are the average ± the standard deviation of three repeated runs.

The sample with 30 wt % ionomer had the lowest *C*_DL_ (35 ± 2 μF; Figure 2b), which increased with decreasing ionomer content to 50 ± 3, 54 ± 4, and 56 ± 8 μF for the samples with 10, 5, and 1 wt % ionomer, respectively (Figure 2b). This result suggests that as the ionomer content decreased, there were fewer active sites on the NiFe_2_O_4_ catalyst that were blocked by the ionomer, either via adsorption of the ionomer to catalytic active sites or via the ionomer physically encapsulating and electronically isolating these sites. As the ionomer was removed from the catalyst layer, however, the *C*_DL_ decreased slightly to 47 ± 3 μF (0 wt %; Figure 2b), caused by large particle agglomeration and decreased effective surface area.

The role of the ionomer in active site utilization was further probed via analysis of the kinetic overpotential trends, shown in Figure 2c and Table 1. Kinetic overpotentials were determined by extrapolating Tafel parameters from high frequency resistance (HFR)-free polarization curves across the entire tested current density regime and subtracting these from the equilibrium potential. We note that samples with different ionomer contents had similar values for HFR (Table 1 and Figure S1a, SI). With no ionomer, the kinetic overpotential was high at 521 ± 2 mV. As ionomer was incorporated, the kinetic overpotential dropped; at 10 5, and 1 wt % ionomer, the kinetic overpotential dropped to 456 ± 16, 459 ± 5, and 480 ± 5 mV, respectively. As the ionomer content increased further, however, the kinetic overpotential increased significantly to 491 ± 15 for the 30 wt % Versogen sample. This is consistent with trends in Tafel slope, which was lowest for samples with intermediate ionomer content (Table 1 and Figure S1b, SI). These results indicate that cell kinetics are improved at intermediate ionomer contents, where catalyst utilization and effective surface areas are increased due to decreased catalyst agglomeration in inks, discussed next.

The role of the ionomer in catalyst agglomeration was examined using DLS measurements (Figure 2d) and gravitational settling experiments (Figure S2, SI). DLS measurements utilize Brownian diffusion of particles, probed by measuring fluctuations in the intensity of light scattering, to determine the particle size and the distribution of dilute colloidal particle dispersions.^24^ Such experiments have been used to study PEMWE and PEMFC inks with Nafion,^1,2,25,26^ but, to the best of our knowledge, similar experiments have not been demonstrated for AEMWE inks with Versogen ionomer.

The sample with 0 wt % Versogen had large agglomerate populations spanning between ∼700–3000 nm (Figure 2d) with a mean *z*-average diameter of 1251 ± 109 nm. This value was significantly larger than the average size of ∼60 nm provided by the supplier, indicating significant agglomeration of catalyst particles. Upon the addition of just 1 wt % of ionomer, the size of the agglomerate populations dropped significantly, with populations ranging between ∼80–340 nm and ∼340–1700 nm, as can be seen from their bimodal intensity distribution (Fig. 2d). As the ionomer content in the inks was increased further to 5, 10, and 30 wt %, the size of the agglomerate populations remained small relative to the 0 wt % ionomer case, ranging between ∼40 and 2000 nm (Figure 2d). These results suggest the addition of ionomer can help slow the agglomeration of particles, possibly via repulsive forces from adsorbed ionomer resisting the van der Waals attraction between particles, similar to how Nafion has been shown to improve stability for carbon black inks,^25,26^ or via electrostatic and steric stabilization mechanisms, similar to how polyelectrolytes stabilize colloidal particles.^27^ This stabilization of catalyst inks by the addition of ionomer can be further seen in gravitational settling results (Figure S2, SI), where the rapid sedimentation observed for samples with no ionomer was significantly slowed by the addition of ionomer. Additional discussion of the ink characterization results, including polydispersity and gravitational settling data, can be found in Section S5 and Figures S3 and S4 (SI).

**Figure 3:**
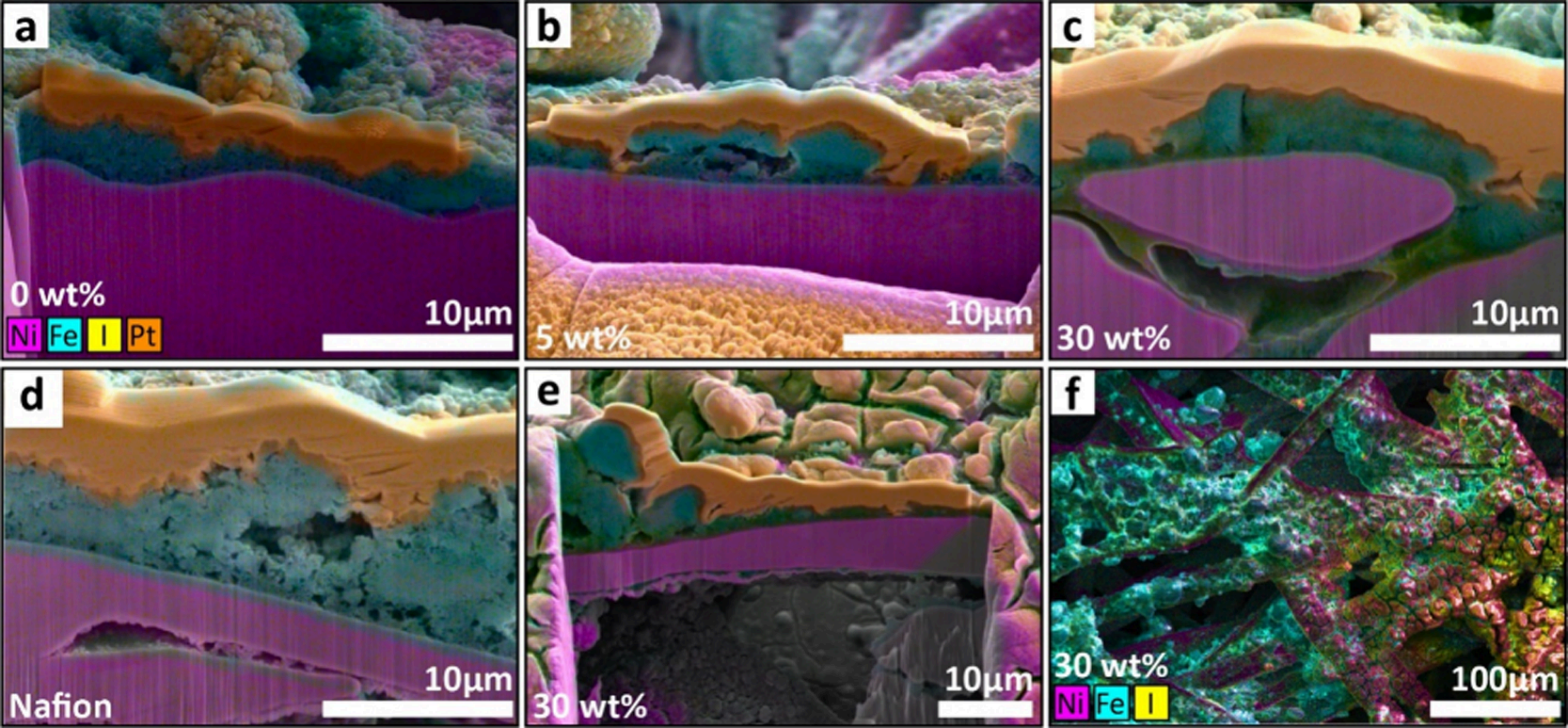
Cross sectional (a–e) and top down (f) SEM microscopy results for samples with 0 wt % Versogen (a), 5 wt % Versogen (b), 10 wt % Nafion (d), and 30 wt % Versogen (c, e, f). (a–e) EDX map showing the Ni, Fe, I, and Pt signals overlaid on secondary electron images. (f) EDX map of the Ni, Fe, and I signal for the 30 wt % Versogen sample. The ionomer is represented by the I signal in yellow, the catalyst phase is shown in cyan (Fe) and magenta (Ni), the Ni mesh transport layer fibers are shown in magenta, and the protective Pt coating added over the catalyst layer before FIB cross sectioning is shown in orange.

The effects of ionomer content on catalyst layer structure were further studied via SEM-EDX imaging of the catalyst layers; representative images of these layers are shown in Figure 3. At all ionomer contents for the Versogen ionomer, the pristine catalyst layer thickness was approximately 5 μm. Catalyst layers with different ionomer contents, however, exhibited qualitative differences in porosity and homogeneity. With no ionomer, (0 wt %, Figure 3a) some porosity was observed. The porosity increased at 5 wt % ionomer, where larger spaces between particle agglomerates were evident (Figure 3b). As ionomer content increased to 30 wt % (Figure 3c), the catalyst layer and agglomerates became dense relative to lower ionomer content samples. The sample with 10 wt % Nafion (Figure 3d), in contrast, exhibited the highest porosity compared to the samples with Versogen ionomer; this sample had a catalyst layer thickness of approximately 10 μm despite similar loadings, and pockets of air were clear throughout the catalyst layer. The SEM images also showed significant heterogeneity in the 30 wt % Versogen sample. This sample had regions of dense, agglomerated catalyst particles (leftmost side of the catalyst layer, Figure 3e) as well as regions with a very thin catalyst layer (rightmost side, Figure 3e). Areas of thick ionomer coverage were also evident, as can be seen by the strong yellow I signal at the rightmost side of Figure 3f; this increase in ionomer coverage corresponds to morphological changes in the catalyst coating seen on the right side of Figure 3e. This heterogeneity can be seen even more clearly in top-down images, as shown in Figure 6a–c, and is consistent with significant blocking of catalyst active sites by ionomer and the observed activity trends.

#### The Role of the Ionomer in Ion Conduction

Anodes constructed with K^+^-exchanged Nafion as the ionomer phase were used to study the role of the ionomer in ion conduction through the catalyst layer, and to evaluate the necessity of the ionomer having an anion-exchange functionality. The results for the sample with 10 wt % K^+^-exchanged Nafion had the lowest average cell overpotential (highest activity) of all MEAs tested (Figure 2a and Table 1), at 617 ± 5 nm. This result demonstrates that an anion-exchange functionality is not required of the ionomer in the catalyst layer when supporting electrolyte is employed.

**Figure 4:**
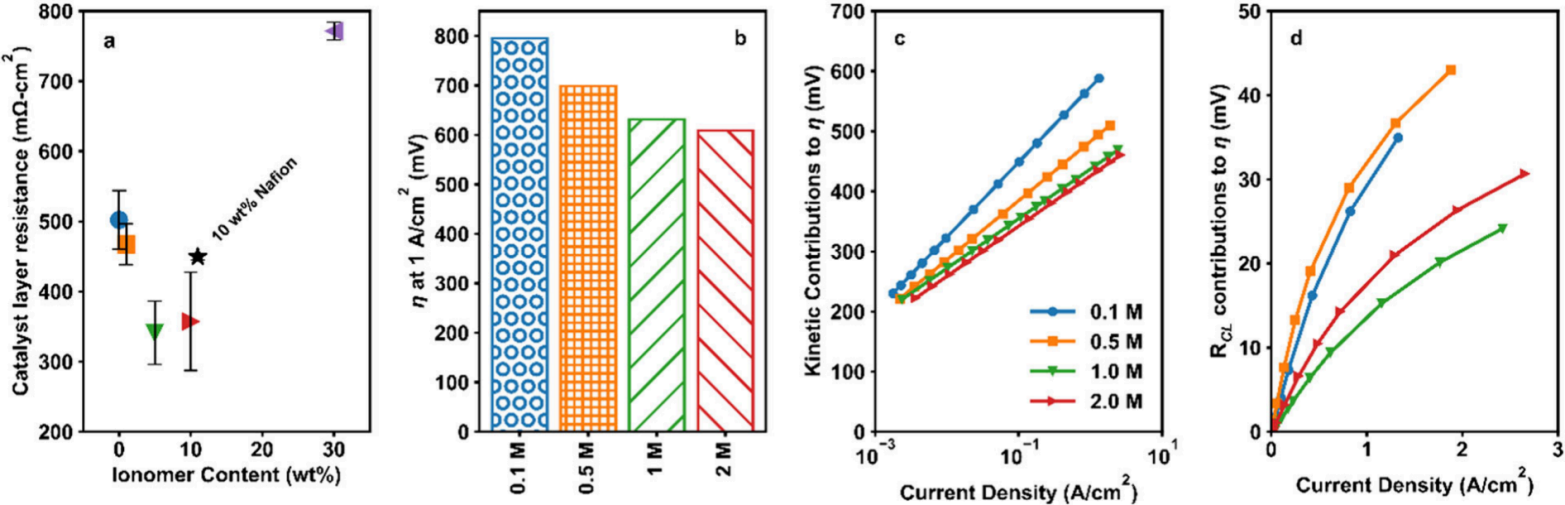
(a) Catalyst layer resistances for samples with different Versogen and Nafion ionomer contents, shown as the average and standard deviation of three repeated runs. (b) Overpotentials at 1 A/cm^2^, (c) kinetic contributions to overpotentials, and (d) catalyst layer resistance contributions to overpotentials for cells with 10 wt % Versogen in the anode catalyst layers and four different electrolyte concentrations: 0.1 M, 0.5 M, 1 M, and 2M.

The role of the ionomer in disrupting electronic and ionic conductivity pathways throughout the catalyst layer was studied via analysis of the *R*_CL_ trends (Fig. 4a). *R*_CL_ is related to through- and in-plane resistive losses within the catalyst layer itself, including resistances to both electronic and ionic transport.^21,28^ Past works have shown that the *R*_CL_ in AEMWEs is sensitive to disruptions in electronic pathways through the catalyst layer, such as by poor catalyst conductivity, poor catalyst-catalyst contact, and/or poor catalyst-transport layer contact.^29^ Values for *R*_CL_ were determined from non-Faradaic EIS measurements at 1.25 V and calculated using transmission line theory for porous electrodes,^21,28^ as discussed in our previous work.^18^

With no ionomer in the catalyst layer, the *R*_CL_ was 502 ± 42 mΩ-cm^2^ (Figure 4a). With the addition of 1 wt % Versogen ionomer, the average *R*_CL_ dropped slightly to 467 ± 29 mΩ-cm^2^. As the Versogen ionomer content continued to increase, the average *R*_CL_ dropped dramatically to 341 ± 45 and 357 ± 70 mΩ-cm^2^ for 5 and 10 wt % ionomer contents, respectively (Figure 4a). Larger particle agglomerates for the 0 wt % sample (Figure 2d, discussed above) likely contributed to inhibited catalyst-catalyst contact and an increase in electronic resistances throughout the catalyst layer for the lower ionomer content samples, leading to the improved *R*_CL_s at intermediate ionomer contents. As the Versogen ionomer content was increased further, however, this trend reversed and the *R*_CL_ dramatically increased to 771 ± 13 mΩ-cm^2^, more than double the *R*_CL_ at intermediate ionomer content. This result is consistent with the ionomer blocking active sites and limiting electronically insulating particles at high ionomer contents, consistent with the electrochemical performance trends.

*R*_CL_ trends for the Versogen samples vs the Nafion samples provide further insights into the ionic conductivity contributions to *R*_CL_. The sample with 10 wt % Nafion had an *R*_CL_ of 450 ± 5 mΩ-cm^2^, slightly lower than that of sample with no ionomer, even though both samples lacked an anion-conducting functionality in the catalyst layer. This result suggests that mixed ionic and electronic limitations impacted the 0 wt % sample. The significantly lower catalyst layer resistances for the 10 wt % Versogen sample vs. the 10 wt % Nafion sample point to ionic resistances in the absence of ionomer, though these were not found to impact catalytic activity trends (low η of Nafion vs Versogen samples, Figure 2a). This suggests that electronic and ionic limitations both contribute to overall *R*_CL_s.

The role of the ionomer in ion conduction was further probed via changes to the electrolyte concentration (0.1 to 2.0 M) with a fixed amount of the Versogen ionomer in the catalyst layer (10 wt %); the results are shown in Figure 4b–d. Improved performance was observed with increasing electrolyte concentration, consistent with literature expectations (Figure 4b).^30,31^ Notably, there were marginal improvements in the performance when increasing from 1 to 2 M, compared to the difference observed between 1 M and lower electrolyte concentrations, suggesting that 1 M may provide sufficient ionic conductivity to support cell needs. These performance differences appear to be driven by kinetic and *R*_CL_ changes. Kinetic losses decreased with increased electrolyte concentration (Figure 4c). This is expected, as at higher electrolyte concentrations there is an increased availability of reacting species (OH^–^) and a predicted increase of accessibility of these reactants to catalytic active sites due to increased ionic transport networks provided by the supporting electrolyte. These improvements in kinetic losses, however, were marginal above 1 M KOH, suggesting that 1 M KOH provides sufficient concentrations of the OH- reactant and facile ion transport pathways. *R*_CL_ losses also decreased with increased electrolyte concentration (Figure 4d), with the most dramatic improvement occurring between 0.5 and 1 M KOH. The RCL for 0.1 and 0.5 M KOH were similar and considered equivalent within the typical experimental error for MEA testing. These results again suggest that 1 M KOH is sufficient to provide transport pathways and reduce resistances in the catalyst layer.

#### The Role of the Ionomer in Catalyst Layer Stability

**Figure 5:**
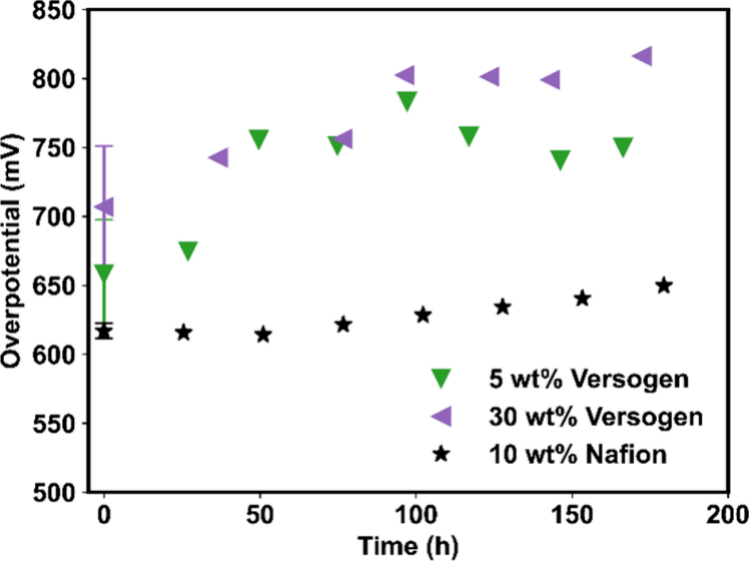
Durability testing results for 5 wt % Versogen, 30 wt % Versogen, and 10 wt % Nafion. Beginning of life values are taken from the average of three repeated runs, as shown Figure 2a. Samples were held at 1.8 V for 200 h, with polarization curves taken every two hours. Plotted overpotentials are calculated from potentiostatic polarization curves at the time intervals indicated and were normalized to the averaged beginning-of-life.

Samples with different ionomer contents were tested over 200 h to assess the role of ionomer content in AEMWE durability. The results of these tests are plotted in Figure 5 as the overpotential at 1 A/cm^2^ for approximately every 24 h of the 200-h test. For the 5 wt % sample (green downfacing triangles, Figure 5), most of the activity loss occurred within the first 48 h of testing. At the beginning of life, the overpotential was 658 ± 39 mV, and this increased to 755 mV within 48 h (an initial degradation rate of 2.0 mV/h). The overpotential then remained at 755 ± 13 mV for the remainder of the 200-h test, an increase of +15% or a total degradation rate of 0.5 mV/h. The sample with 30 wt % ionomer (purple left-facing triangles, Figure 5) exhibited similar overall activity loss (an increase of 13% or a degradation rate of 0.5 mV/h over the 200-h test), though this degradation occurred more gradually. The sample with 10 wt % Nafion (black stars, Figure 5), in contrast, exhibited the most stable performance over the 200-h testing. At the beginning of life, the overpotential was 617 ± 5 mV and this increased to 638 mV after 200 h, an increase of 3.4% or a total degradation rate of 0.1 mV/h. Such degradation rates, however, are two orders of magnitude higher than they need to be to compete with PEMWE; the technical target for PEMWE degradation rates is 2.0 μV/h.^32^ These results demonstrate that the identity of the ionomer, its chemical stability, and its quantity all impact the rate of degradation. Possible degradation routes were probed with microscopic and spectroscopic techniques, as discussed in the following paragraphs.

**Figure 6:**
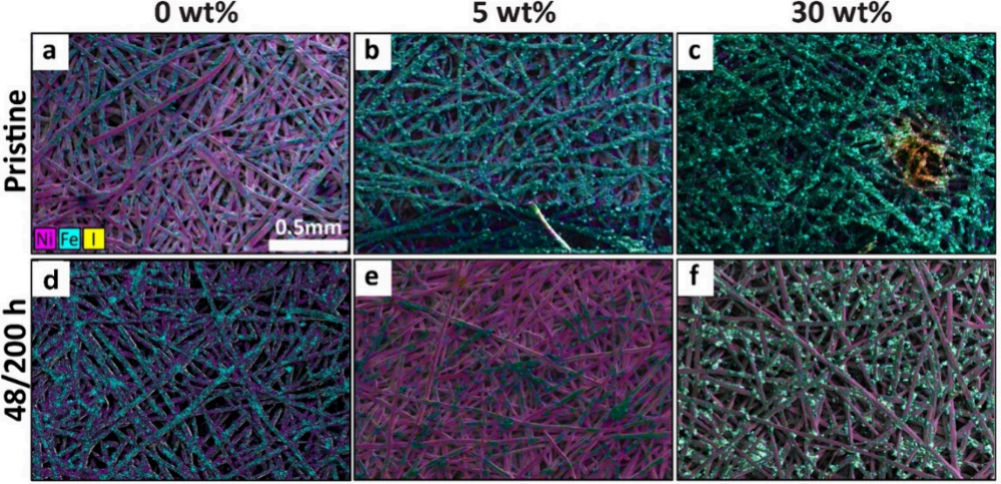
Top-down SEM-EDX with Ni Fe and I maps overlaid on images of untested (a–c) and long-term tested (48–200 h; d–f) samples with 0, 5, and 30 wt % Versogen Ni, Fe, and I elements are shown using magenta, cyan, and yellow at 500× for all images.

The role of the ionomer in catalyst adhesion was probed via SEM-EDX images of pristine and long-term tested samples at different ionomer contents. All samples showed thinning and loss of porosity in the catalyst layer after testing, as seen in post-mortem cross sections of the anodes (Figure S5, SI). In the top-down images (Figure 6), the coverage of the Ni mesh transport layer fibers (shown in magenta) by the NiFe_2_O_4_ catalyst (represented by the Fe signal in cyan) between the pristine (Figure 6a–c) and tested samples (Figure 6d–f) was found to vary based on the ionomer content in the catalyst layer. For the 0 and 5 wt % samples, significant catalyst loss (apparent from changes to the Fe signals in the EDX map) can be clearly seen between the images for the pristine and tested samples (Figure 6a vs d for 0 wt % and Figure 6b vs Figure 6e for 5 wt %, respectively). For the 30 wt % sample, in contrast, the Fe signals were still strongly evident after testing (Figure 6c vs 6f), indicating the retention of catalyst particles on the surface. Some catalyst loss was also visually observed for the samples with 10 wt % Nafion; these results are shown in Figure S6, SI.

To quantitatively compare the catalyst loss between 5 and 30 wt % samples, XRF analysis of the samples before and after testing was performed to quantify the change in Fe signal in mg/cm^2^. Quantification using the Fe signal was necessary because the Ni signal from the Ni mesh transport layer used as the substrate for all anode electrodes interfered with the quantification of Ni in the catalyst. The 5 wt % sample exhibited a 63% loss of Fe loading in the first 24 h, and 78% total Fe loss after 200 h (Figure S7, SI and Table S1). These losses were greater than those observed for the 30 wt % sample, which exhibited a 20 wt % loss in Fe loading in the first 24 h, and a 41% total Fe loss after 200 h (Figure S7, SI and Table S1). This result suggests that an increased quantity of the ionomer phase can help ensure catalyst adhesion to the substrate and follows the observed trends in durability testing, which showed lower initial degradation rates for the 30 wt % vs 5 wt % sample (Figure 5). Note that some detached catalyst particles may have been transferred to and partially embedded in the membrane during testing or disassembly; indeed, post-test XRF measurements of the tested membranes revealed the presence of Fe (Table S2, SI). Such movement of particles from the transport layer to the membrane may not necessarily lead to degradation, as these catalyst particles are likely still in contact with the transport layer and therefore still electronically connected and active.

**Figure 7:**
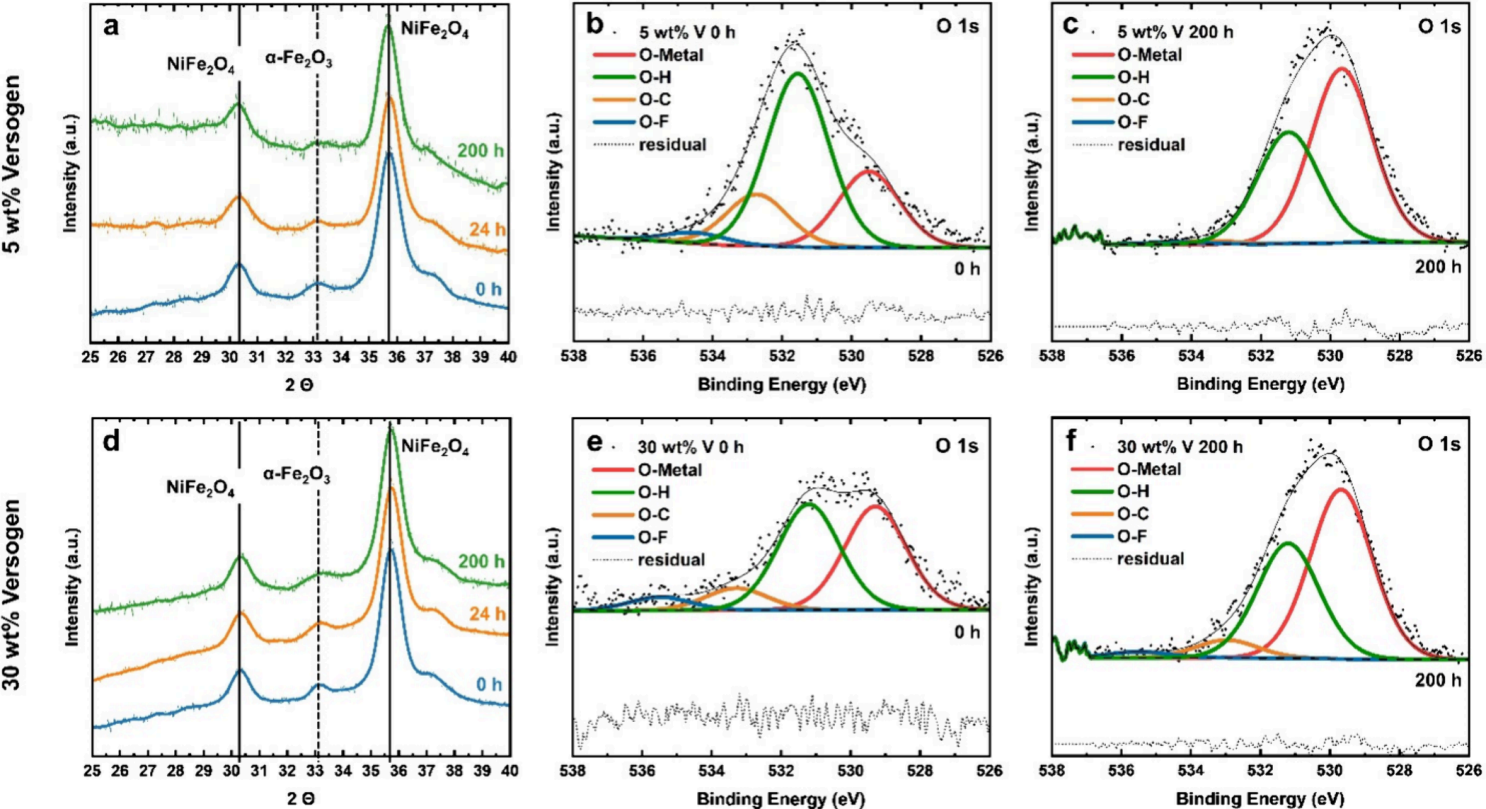
XRD results for 5 wt % (a) and 30 wt % (d) Versogen samples before and after testing. XPS results for 5 wt % Versogen (b, e) and 30 wt % Versogen (c, f) before and after 200 h durability testing.

To further investigate the role of the ionomer in catalyst layer stability and to understand this observed Fe loss, ex-situ XRD and ICP-MS analyses were employed. Figure 7a, d show the pristine (black), 24 h tested (orange), and 200 h tested (blue) XRD results for the 5 wt % (Figure 7a) and 30 wt % (Figure 7d) ionomer samples. The peak at 2θ = 33.2°, corresponding to the α-Fe_2_O_3_ phase (JCPDS card 39–1346), decreased with increasing time-on-stream relative to the peaks associated with NiFe_2_O_4_ (2θ = 30.4°, 35.7°; JCPDS card 86-2267). For the 5 wt % sample, there was a 47% loss in the α-Fe_2_O_3_ peak relative to NiFe_2_O_4_, and in the 30 wt % sample this loss was 19% (details in Section S7, SI). These results suggest that catalyst loss is selective towards the α-Fe_2_O_3_ phase, which is a known contaminant in this commercial material. This is consistent with ICP-MS measurements, where selective Fe loss was observed (Figure S8, SI).

Changes to the ionomer before and after testing were evaluated using ex-situ XPS analysis of O 1s, C 1s, N 1s, and F 1s spectra of pristine and tested catalyst layers. N and F are of interest as constituents of the functional group and backbone of the polymer, respectively. Unfortunately, the N and F content in the polymer were relatively low, such that no signal was observed in the N 1s and F 1s spectra for the pristine or tested 5 wt % sample (Figure S9). Both were observable for the pristine 30 wt % sample, however, with a peak at 402 eV in the N 1s spectrum corresponding to the ammonium functional group reported for Versogen^33^ and a peak at 687 eV in the F 1s spectrum corresponding to the organic C–F moiety in the backbone (Figure S10).^34^ After the 200-h test, the signal decreased significantly, although the F 1s peak remained at 687 eV. This indicates loss or possible degradation of the polymer during testing.

Lindquist et al. similarly observed a loss of N 1s and F 1s signal for the Versogen PiperION ionomer after 20 h testing in deionized water and 0.1 M KOH, which they attributed to degradation of the polymer backbone and cation functional groups. For their testing in DIW, they further observed an accompanying increase in the peak between 288 and 289 eV in the C 1s spectra, which they attributed to carbonyl and/or ester group formation, and stated that this peak in conjunction with the loss of N 1s and F 1s signal indicated oxidation of the Versogen polymer.^7^ No increase in the peak at 289 eV was observed for the C 1s spectra of either Versogen sample (5 or 30 wt %) tested in this work, and we cannot conclusively say that the ionomer loss was due to oxidation. However, there may be other polymer degradation routes occurring; our work utilized 1 M KOH supporting electrolyte, and the high pH may promote different (in)stabilities versus the 0.1 M KOH case from the work of Lindquist et al. Additionally, our work employed a NiFe_2_O_4_ anode catalyst compared to the IrO_2_ (pure water) and Co_3_O_4_ (0.1 M) catalysts utilized in the work by Lindquist et al., which may have facilitated different degradation mechanisms for the Versogen polymer. Polymer loss may also have occurred via physical routes, i.e., via liquid uptake and swelling and leading to catalyst layer delamination.

The relative loss of ionomer versus catalyst after testing was evaluated and quantified using ex-situ XPS analysis of O 1s spectra of pristine and tested catalyst layers. The O 1s spectra were deconvoluted into O-metal (red, ∼529.5 eV) and O–F (blue, ∼535 eV) peaks, as well as peaks at ∼531.5 and 532.5 eV that are tentatively assigned to O–H and O=C species (Figure 7b,c,e,f).^34^ The more clearly assignable metal oxide and hydroxide peaks were compared to the O–F peaks as representatives of the catalyst and the polymer, respectively. For the pristine 5 and 30 wt % samples, the ratios of O–F/O(H)–M were 4 and 7% (Figure 7b,e). After 200 h of testing, these ratios decreased to 1 and 2% for the 5 and 30 wt % samples, respectively, confirming the loss of polymer and generally higher polymer content for the 30 wt % samples (Figure 7c,f). For the durability test with 10 wt % Nafion in the anode catalyst layer, the XPS results indicated that there was some loss of polymer (evidenced by a decrease in the S signal and in the O–F and O–S bonds, Figure S11, SI). These results are discussed in more detail in Section S9 of the SI. While the XRF and XRD results discussed above indicate differences in catalyst loss at different ionomer contents, the XPS analysis shows that Versogen ionomer loss and/or degradation at the surface of the catalyst layer is significant after testing. These stability testing results indicate that cell losses were likely driven largely by catalyst detachment and loss, driven by a combination of insufficient polymer/insufficient adhesion as well as polymer loss. A sophisticated understanding of cell losses over time, including additional details on the mechanisms of catalyst loss and ionomer degradation, are the subject of future work in our group. Additionally, future analysis of the Ni/Fe 2p spectra would be helpful to understand changes in metal oxidation state and the formation of (oxy)hydroxide species that likely are observed in the O 1s spectra.

## Conclusions

In this work, the role of the ionomer in supporting electrolyte-fed AEMWEs was systematically studied by linking trends in cell activities with catalyst layer microstructure, ink stability, and kinetic and electrochemical signatures (e.g., Tafel kinetics, catalyst layer resistances, and effective surface areas) for anode catalyst layers with varying amounts of Versogen and Nafion polymers. The results show that the ionomer is not required as an ion conductor when AEMWEs are operated with supporting electrolyte. Instead, the ionomer was found to play an important role in anode catalyst layer structure and active site accessibility. Specifically, intermediate ionomer contents (5–10 wt %) were found to provide the highest cell current densities due to a balance of catalyst layer structure, homogeneity, and stability. Furthermore, the results indicate that celllosses occured at an accelerated rate for anodes with less ionomer in the catalyst layer (i.e., 5 wt %) and for samples with an anion exchange polymer (i.e., Versogen) vs Nafion, and that this loss is concurrent with catalyst detachment and ionomer loss.

Importantly, these results indicate that the polymer used in the catalyst layer does not need to be of the same chemistry as the anion exchange membrane, mitigating limitations caused by ionomer instabilities (such as swelling and oxidation) as has been reported in the literature. Future works that focus on isolating qualities of the polymer phase that can prevent catalyst detachment and are resistant to physical and chemical degradation are desired to help decrease loss rates for AEMWEs. To support preferred catalyst layer structures (i.e., high degree of porosity, homogeneity of catalyst and polymer phases, and uninterrupted transport networks for electrons, ions, liquids, and evolved gases) polymers that do not block or occupy catalyst active sites, but that instead can help facilitate homogeneous dispersion of catalyst and ionomer and facilitate uniform catalyst layers, are likely advantageous. The exploration of the effects of electrolyte concentrations on catalyst layers with polymers with different ion exchange capacities would be an interesting future avenue for this work, including varying electrolyte concentration with Nafion as the ionomer. Additionally, expanding this study to look at cathode catalyst layers, which likely have different needs from the anode, including water transport and management, is desired. Overall, the results of this work can help accelerate the development and deployment of AEMWEs and other electrochemical devices that utilize anion exchange polymers.

## Supporting Information Available

Additional experimental details, results, and analysis (PDF)

## References

[ref1] Khandavalli S.; Park J. H.; Kariuki N. N.; Myers D. J.; Stickel J. J.; Hurst K.; Neyerlin K. C.; Ulsh M.; Mauger S. A. Rheological Investigation on the Microstructure of Fuel Cell Catalyst Inks. ACS Appl. Mater. Interfaces 2018, 10 (50), 43610–43622. 10.1021/acsami.8b15039. 30525374

[ref2] Khandavalli S.; Park J. H.; Kariuki N. N.; Zaccarine S. F.; Pylypenko S.; Myers D. J.; Ulsh M.; Mauger S. A. Investigation of the Microstructure and Rheology of Iridium Oxide Catalyst Inks for Low-Temperature Polymer Electrolyte Membrane Water Electrolyzers. ACS Appl. Mater. Interfaces 2019, 11 (48), 45068–45079. 10.1021/acsami.9b14415. 31697470

[ref3] Faid A. Y.; Xie L.; Barnett A. O.; Seland F.; Kirk D.; Sunde S. Effect of Anion Exchange Ionomer Content on Electrode Performance in AEM Water Electrolysis. International Journal of Hydrogen Energy 2020, 45 (53), 28272–28284. 10.1016/j.ijhydene.2020.07.202.

[ref4] Cossar E.; Barnett A. O.; Seland F.; Safari R.; Botton G. A.; Baranova E. A. Ionomer Content Optimization in Nickel-Iron-Based Anodes with and without Ceria for Anion Exchange Membrane Water Electrolysis. J. Power Sources 2021, 514, 230563 10.1016/j.jpowsour.2021.230563.

[ref5] Volk E. K.; Kreider M. E.; Kwon S.; Alia S. M. Recent Progress in Understanding the Catalyst Layer in Anion Exchange Membrane Electrolyzers - Durability, Utilization, and Integration. EES. Catal. 2024, 2, 109 10.1039/D3EY00193H.

[ref6] Liu J.; Kang Z.; Li D.; Pak M.; Alia S. M.; Fujimoto C.; Bender G.; Kim Y. S.; Weber A. Z. Elucidating the Role of Hydroxide Electrolyte on Anion-Exchange-Membrane Water Electrolyzer Performance. J. Electrochem. Soc. 2021, 168 (5), 054522 10.1149/1945-7111/ac0019.

[ref7] Lindquist G. A.; Gaitor J. C.; Thompson W. L.; Brogden V.; Noonan K. J. T.; Boettcher S. W. Oxidative Instability of Ionomers in Hydroxide-Exchange-Membrane Water Electrolyzers. Energy Environ. Sci. 2023, 16 (10), 4373–4387. 10.1039/D3EE01293J.

[ref8] Henkensmeier D.; Najibah M.; Harms C.; Žitka J.; Hnát J.; Bouzek K. Overview: State-of-the Art Commercial Membranes for Anion Exchange Membrane Water Electrolysis. J. Electrochem. Energy Convers. Storage 2021, 18 (2), 024011 10.1115/1.4047963.

[ref9] Chen B.; Mardle P.; Holdcroft S. Probing the Effect of Ionomer Swelling on the Stability of Anion Exchange Membrane Water Electrolyzers. J. Power Sources 2022, 550, 232134 10.1016/j.jpowsour.2022.232134.

[ref10] Huang G.; Mandal M.; Hassan N. U.; Groenhout K.; Dobbs A.; Mustain W. E.; Kohl P. A. Ionomer Optimization for Water Uptake and Swelling in Anion Exchange Membrane Electrolyzer: Oxygen Evolution Electrode. J. Electrochem. Soc. 2020, 167 (16), 164514 10.1149/1945-7111/abcde3.

[ref11] Mardle P.; Chen B.; Holdcroft S. Opportunities of Ionomer Development for Anion-Exchange Membrane Water Electrolysis. ACS Energy Lett. 2023, 8, 3330–3342. 10.1021/acsenergylett.3c01040.

[ref12] Mayerhöfer B.; Speck F. D.; Hegelheimer M.; Bierling M.; Abbas D.; McLaughlin D.; Cherevko S.; Thiele S.; Peach R. Electrochemical- and Mechanical Stability of Catalyst Layers in Anion Exchange Membrane Water Electrolysis. International Journal of Hydrogen Energy 2022, 47 (7), 4304–4314. 10.1016/j.ijhydene.2021.11.083.

[ref13] Krivina R. A.; Lindquist G. A.; Beaudoin S. R.; Stovall T. N.; Thompson W. L.; Twight L. P.; Marsh D.; Grzyb J.; Fabrizio K.; Hutchison J. E.; Boettcher S. W. Anode Catalysts in Anion-Exchange-Membrane Electrolysis without Supporting Electrolyte: Conductivity, Dynamics, and Ionomer Degradation. Adv. Mater. 2022, 34 (35), 2203033 10.1002/adma.202203033. 35790033

[ref14] Ghoshal S.; Pivovar B. S.; Alia S. M. Evaluating the Effect of Membrane-Ionomer Combinations and Supporting Electrolytes on the Performance of Cobalt Nanoparticle Anodes in Anion Exchange Membrane Electrolyzers. J. Power Sources 2021, 488, 229433 10.1016/j.jpowsour.2020.229433.

[ref15] Li D.; Matanovic I.; Lee A. S.; Park E. J.; Fujimoto C.; Chung H. T.; Kim Y. S. Phenyl Oxidation Impacts the Durability of Alkaline Membrane Water Electrolyzer. ACS Appl. Mater. Interfaces 2019, 11 (10), 9696–9701. 10.1021/acsami.9b00711. 30811171

[ref16] Matanovic I.; Kim Y. S. Electrochemical Phenyl Oxidation: A Limiting Factor of Oxygen Evolution Reaction in Water Electrolysis. Current Opinion in Electrochemistry 2023, 38, 101218 10.1016/j.coelec.2023.101218.

[ref17] Koch S.; Heizmann P. A.; Kilian S. K.; Britton B.; Holdcroft S.; Breitwieser M.; Vierrath S. The Effect of Ionomer Content in Catalyst Layers in Anion-Exchange Membrane Water Electrolyzers Prepared with Reinforced Membranes (Aemion+^TM^). J. Mater. Chem. A 2021, 9 (28), 15744–15754. 10.1039/D1TA01861B.

[ref18] Volk E. K.; Kwon S.; Alia S. M. Catalytic Activity and Stability of Non-Platinum Group Metal Oxides for the Oxygen Evolution Reaction in Anion Exchange Membrane Electrolyzers. J. Electrochem. Soc. 2023, 170 (6), 064506 10.1149/1945-7111/acd605.

[ref19] Fairley N.; Fernandez V.; Richard-Plouet M.; Guillot-Deudon C.; Walton J.; Smith E.; Flahaut D.; Greiner M.; Biesinger M.; Tougaard S.; Morgan D.; Baltrusaitis J. Systematic and Collaborative Approach to Problem Solving Using X-Ray Photoelectron Spectroscopy. Applied Surface Science Advances 2021, 5, 100112 10.1016/j.apsadv.2021.100112.

[ref20] Biesinger M. C.; Payne B. P.; Grosvenor A. P.; Lau L. W. M.; Gerson A. R.; Smart R. St. C. Resolving Surface Chemical States in XPS Analysis of First Row Transition Metals, Oxides and Hydroxides: Cr, Mn, Fe, Co and Ni. Applied Surface Science 2011, 257 (7), 2717–2730. 10.1016/j.apsusc.2010.10.051.

[ref21] Padgett E.; Bender G.; Haug A.; Lewinski K.; Sun F.; Yu H.; Cullen D. A.; Steinbach A. J.; Alia S. M. Catalyst Layer Resistance and Utilization in PEM Electrolysis. J. Electrochem. Soc. 2023, 170 (8), 084512 10.1149/1945-7111/acee25.

[ref22] McCrory C. C. L.; Jung S.; Ferrer I. M.; Chatman S. M.; Peters J. C.; Jaramillo T. F. Benchmarking Hydrogen Evolving Reaction and Oxygen Evolving Reaction Electrocatalysts for Solar Water Splitting Devices. J. Am. Chem. Soc. 2015, 137 (13), 4347–4357. 10.1021/ja510442p. 25668483

[ref23] Trasatti S.; Petrii O. A. Real Surface Area Measurements in Electrochemistry. Journal of Electroanalytical Chemistry 1992, 327 (1), 353–376. 10.1016/0022-0728(92)80162-W.

[ref24] Ross Hallett F. Scattering and Particle Sizing, Applications. In Encyclopedia of Spectroscopy and Spectrometry; Lindon J. C., Ed.; Elsevier: Oxford, 1999; pp 2067–2074 10.1006/rwsp.2000.0273.

[ref25] Dixit M. B.; Harkey B. A.; Shen F.; Hatzell K. B. Catalyst Layer Ink Interactions That Affect Coatability. J. Electrochem. Soc. 2018, 165 (5), F264 10.1149/2.0191805jes.

[ref26] Shukla S.; Bhattacharjee S.; Weber A. Z.; Secanell M. Experimental and Theoretical Analysis of Ink Dispersion Stability for Polymer Electrolyte Fuel Cell Applications. J. Electrochem. Soc. 2017, 164 (6), F600 10.1149/2.0961706jes.

[ref27] Napper D. H. Polymeric Stabilization of Colloidal Dispersions: Colloid Science (London, England: 1976); 3; Academic Press: London, 1983.

[ref28] Huang J.; Gao Y.; Luo J.; Wang S.; Li C.; Chen S.; Zhang J. Editors’ Choice—Review—Impedance Response of Porous Electrodes: Theoretical Framework, Physical Models and Applications. J. Electrochem. Soc. 2020, 167 (16), 166503 10.1149/1945-7111/abc655.

[ref29] Kreider M. E.; Yu H.; Osmieri L.; Parimuha M. R.; Reeves K. S.; Marin D. H.; Hannagan R. T.; Volk E. K.; Jaramillo T. F.; Young J. L.; Zelenay P.; Alia S. M. Understanding the Effects of Anode Catalyst Conductivity and Loading on Catalyst Layer Utilization and Performance for Anion Exchange Membrane Water Electrolysis. ACS Catal. 2024, 14, 10806–10819. 10.1021/acscatal.4c02932. 39050897 PMC11264204

[ref30] Lei C.; Yang K.; Wang G.; Wang G.; Lu J.; Xiao L.; Zhuang L. Impact of Catalyst Reconstruction on the Durability of Anion Exchange Membrane Water Electrolysis. ACS Sustainable Chem. Eng. 2022, 10 (50), 16725–16733. 10.1021/acssuschemeng.2c04855.

[ref31] Rossi R.; Taylor R.; Logan B. E. Increasing the Electrolyte Salinity to Improve the Performance of Anion Exchange Membrane Water Electrolyzers. ACS Sustainable Chem. Eng. 2023, 11 (23), 8573–8579. 10.1021/acssuschemeng.3c01245.

[ref32] U.S. Department of Energy Hydrogen and Fuel Cells Technology Office. Technical Targets for Proton Exchange Membrane Electrolysis. Energy.gov. https://www.energy.gov/eere/fuelcells/technical-targets-proton-exchange-membrane-electrolysis (accessed 2023-08-01).

[ref33] Xiao J.; Oliveira A. M.; Wang L.; Zhao Y.; Wang T.; Wang J.; Setzler B. P.; Yan Y. Water-Fed Hydroxide Exchange Membrane Electrolyzer Enabled by a Fluoride-Incorporated Nickel-Iron Oxyhydroxide Oxygen Evolution Electrode. ACS Catal. 2021, 11 (1), 264–270. 10.1021/acscatal.0c04200.

[ref34] National Institute of Standards and Technology. NIST X-Ray Photoelectron Spectroscopy Database, NIST Standard Reference Database Number 20; 2000, 20899 10.18434/T4T88K.

