# Supplementary material for: Role of the Ionomer in Supporting Electrolyte-Fed Anion Exchange Membrane Water Electrolyzers
Source: ACS Electrochem. 2024 Nov 6;1(2):239–48. doi: 10.1021/acselectrochem.4c00061 (PMC11808641; doi:10.1021/acselectrochem.4c00061)
Supplement: Supplementary file 1 — ec4c00061_si_001.pdf [file ec4c00061_si_001.pdf]

Supplementary Information

The role of the ionomer in supporting electrolyte-fed anion exchange membrane water electrolyzers

Emily K. Volk[1], Arielle L. Clauser[2], Melissa E. Kreider[3], Diego D. Soetrisno[4], Sunilkumar Khandavalli[4], Joshua D. Sugar[2], Stephanie Kwon[5*], Shaun M. Alia[3*]

[1]Advanced Energy Systems Graduate Program, Colorado School of Mines, Golden Colorado, 80401, United States

[2]Sandia National Laboratories, Livermore, 94550, California, United States

[3]Chemistry and Nanoscience Center, National Renewable Energy Laboratory, Golden Colorado, 80401, United States

[4]Materials Science Center, National Renewable Energy Laboratory, Golden Colorado, 80401, United States

[5]Department of Chemical and Biological Engineering, Colorado School of Mines, Golden Colorado, 80401, United States

Corresponding author emails: kwon@mines.edu, salia@nrel.gov

Contents

S1. Ion Exchange Procedure for SEM imaging 2
S2. Ion exchange procedure for Nafion polymer 2
S3. Calculation of the thermodynamic potential 3
S4. High Frequency Resistance and Tafel Slope 3
S5. Ink characterization 4
S6. Post-mortem microscopy results 6
S7. XRF and XRD analyses of tested electrodes and membranes 7
S8. ICP-MS analysis of tested electrolytes 9
S9. XPS analysis of tested electrodes 10
References 12

S1. Ion Exchange Procedure for SEM imaging

The Versogen PiperION TP85 ionomer was ion exchanged from $CO3^{2-}$ form to I- from using KI from Sigma Aldrich (99%). The electrodes were placed in a 5.0 M KI solution soak for 72 h. Each sample was soaked in ~10-15 mL of solution in its own sealed container such that the sample was completely covered/saturated and sunk to the bottom of the solution. The soaking samples were then wrapped in lab foil to prevent photodegradation of the KI in solution. After 72 h, the samples were removed and rinsed 3x with DI water. The electrodes were then left to sit in water for 24 hours to remove any remaining K salts. The electrodes were then air dried on and covered in glassine paper for ~24 hours before SEM preparation, which included adhesion to C tape, a C coating of approximately 3-8nm and silver paste to ground the sample to the SEM stub.

S2. Ion exchange procedure for Nafion polymer

Before electrode preparation, a 3.3 wt% K^+ exchanged Nafion solution was prepared by mixing 2.0 mL of 5 wt% Nafion with 1.0 mL of 0.1 M KOH. This solution was stirred overnight to produce the K^+ exchanged Nafion solution. This exchange was performed to ensure that H^+ sites in the Nafion polymer would not interact with and lead to the degradation of the $NiFe_2O_4$ catalyst during electrode preparation.

S3. Calculation of the thermodynamic potential

The thermodynamic potential for electrochemical water splitting was corrected for nonstandard temperature and pressure conditions as follows:[1,2]

$$E_o = 1.229 - \frac{[T - 298.15]\Delta S}{nF} + \frac{RT}{nF}\ln\left(\frac{P_{O_2}P_{H_2}^2}{P_o^3}\right)$$

Where T is the testing temperature in K (in this work, 80 °C), $\Delta S = 2S_{H_2} + S_{O_2} - 2S_{H_2O}$, R is the gas constant (8.314 J/mol K), n is the number of moles of electrons involved in the electrochemical reaction (4 for OER), F is Faraday's constant (96485 C / mol e^-), the partial pressures of O_2 and H_2 (P_{O_2}, P_{H_2}) in Denver are 82.2 kPa, and the standard pressure (P_o) is 101.2 kPa. The thermodynamic potential is therefore **1.178 V** at the studied temperature and pressure conditions.

S4. High Frequency Resistance and Tafel Slope

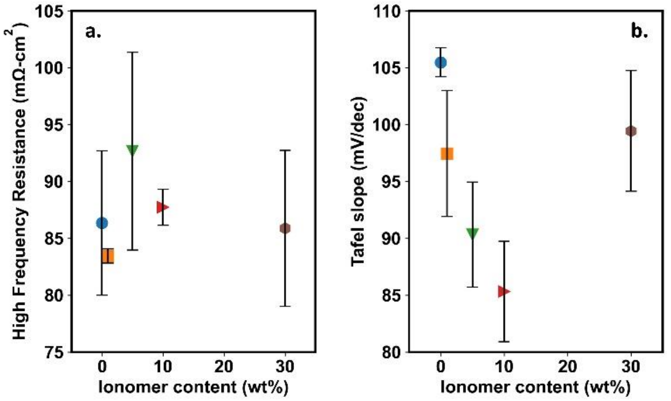

Figure S1. a) Values of the high frequency resistance for samples with different ionomer contents (0 – 30 wt%) and b) values for the Tafel slope for samples with different ionomer contents (0 – 30 wt%).

S5. Ink characterization

Interaction of ionomer with catalyst particles in inks can strongly alter the agglomerate size of the catalyst particles, and, in turn, impact their structure and the degree of ionomer-catalyst interspersion in the catalyst layer; such characteristics are well known to impact device performance by influencing catalyst utilization, electronic and mass transport resistances.

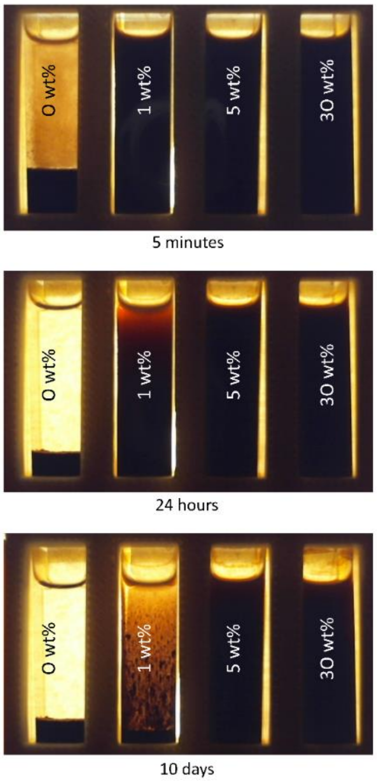

Figure S2. Results for settling experiments for inks with 0, 1, 5, and 30 wt% Versogen ionomers. Inks were of the same composition as those used for anode preparation for all reported cell testing data. Images were collected continuously for a 10-day period and selected images are shown here.

All the inks exhibited a wide size particle distribution with polydispersity indexes (PDI) varying between 0.4 – 0.5, except for 0 wt% ionomer case which was narrow within the measurable range of the DLS technique (Fig S3 in SI). Because of this high polydispersity and the multimodal distribution of agglomerate population of inks with ionomer **(Fig. 2d)**, it is difficult to clearly infer changes in catalyst agglomerate size for ionomer beyond 1 wt% from the DLS data, particularly whether increasing the ionomer concentration continued to cause a decrease in the agglomerate size of the catalyst populations or resulting a maximum size reduction at just 1 wt% ionomer concentration. The ink with 10 wt% Nafion as the ionomer also had a wider size distribution, but the agglomerate populations are smaller compared to 0 wt%

ionomer case, spanning between ~ 70 and 1300 nm, similar to the inks with Versogen as the ionomer.

The trend is clearer from the gravitational settling experiments, comparing the sedimentation rate of the inks as a function of ionomer concentration which depends on the agglomerate size of the particles; the agglomerate size affects the competition between particle diffusion (or Brownian

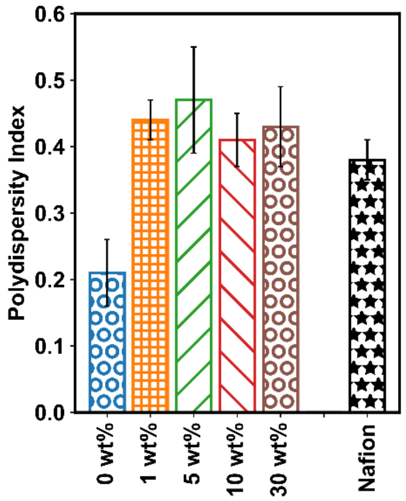

Figure S3. Results for the polydispersity of catalyst inks containing 0-30 wt% Versogen and 10 wt% Nafion ionomers.

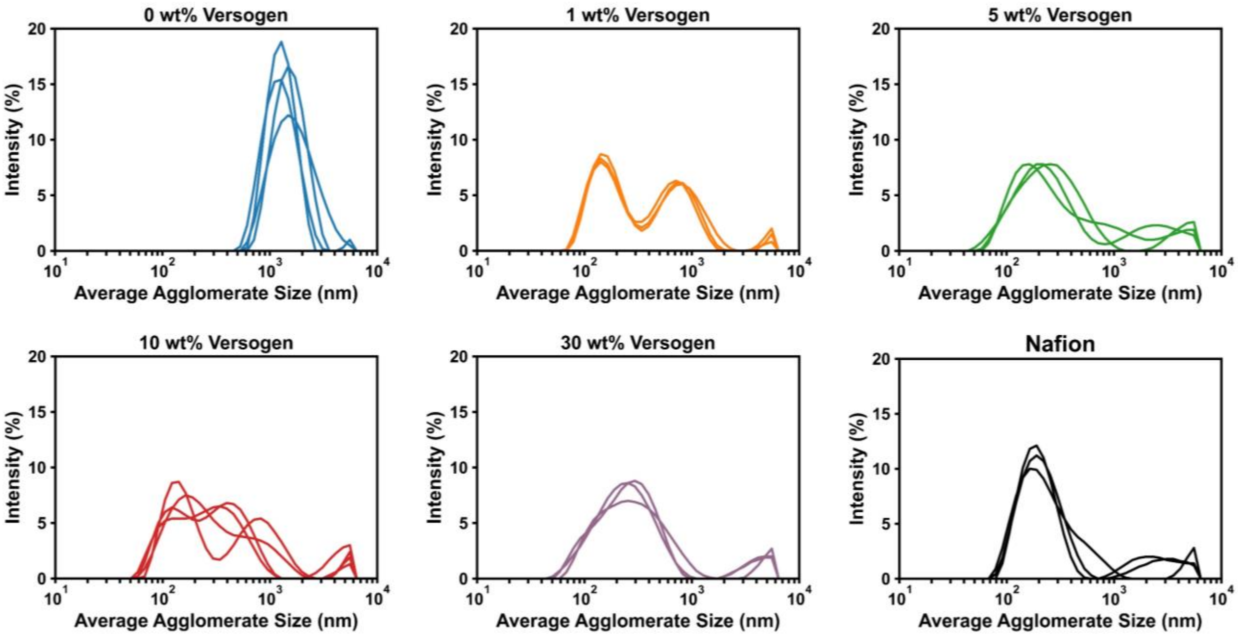

Figure S4. Intensity distribution of particle agglomerate sizes in inks with 0, 1, 5, 10, and 30 wt% Versogen and 10 wt% Nafion.

motion) due to thermal energy and gravity that primarily dictates the sedimentation rate of particles.

The catalyst particles in the 0 wt% ionomer ink settled within the first 5 minutes, but the catalyst particles in the 1 wt% ionomer ink was stable for 24 h before settling began **(Fig. S2)**. No such settling was evident at higher ionomer concentrations, 5 and 30 wt%, over the maximum observed duration of 10 days. The decreasing trend in the settling rate of catalyst particles with increasing ionomer concentration between 0 to 5 wt% indirectly suggests a decrease in the size of agglomerate populations that would retard the sedimentation rate of the particles, which could not be clearly captured from the DLS measurements.

S6. Post-mortem microscopy results

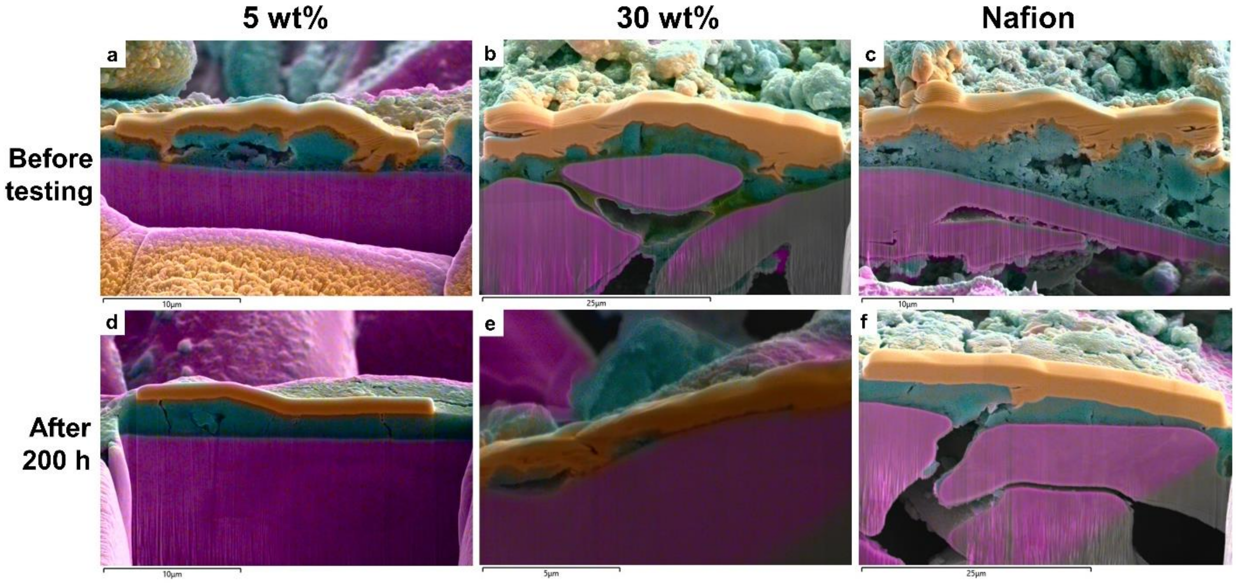

Figure S5. SEM-EDX images for pristine (a-c) and 200-h tested (d-f) catalyst layers with 5 wt% Versogen, 30 wt% Versogen, and 10 wt% Nafion ionomers in the catalyst layer. Orange signal represents the Pt protective overlayer added before cross-sectioning, magenta represents Ni in the transport layer fibers and in the catalyst, and cyan represents Fe in the catalyst.

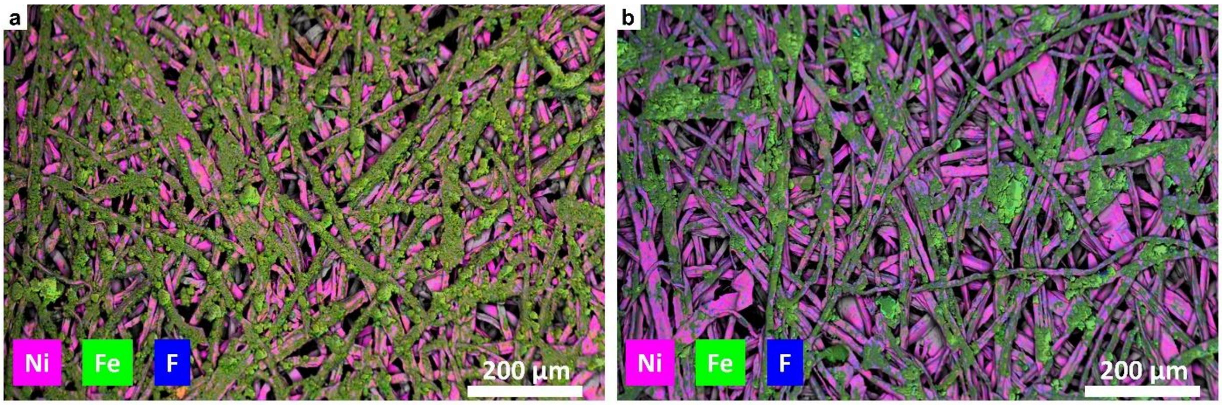

Figure S6. SEM-EDX images for pristine (a) and 200-h tested (b) catalyst layers with 10 wt% Nafion ionomers in the catalyst layer. The magenta represents Ni in the transport layer fibers and in the catalyst, and green represents Fe in the catalyst, and blue represents F in the ionomer.

S7. XRF and XRD analyses of tested electrodes and membranes

The Fe loss for tested anode electrodes was quantified using XRF analysis. Tested anodes were evaluated at 3x 30 s exposures to determine loadings within a standard deviation of 0.05 mg/cm^2, shown in **Table S1**. The percentage loss in Fe loading was quantified from these loading values and is shown in **Fig. S6**. All Fe loadings calculated from XRF and XRD results were from individual tests conducted for different time scales, in contrast with ICP-MS results below which were obtained from the same continuous run.

Table S1. Fe loadings on tested and untested anodes

Sample name	Loading (mg/cm^2)	Standard deviation (mg/cm^2)
0 wt% 0 h	0.324	0.054
0 wt% 24 h	0.178	0.009
0 wt% 48 h	0.151	0.019
5 wt% 0 h	0.425	0.007
5 wt% 24 h	0.158	0.004
5 wt% 200 h	0.095	0.005
30 wt% 0 h	0.750	0.025
30 wt% 24 h	0.600	0.047
30 wt% 200 h	0.439	0.034

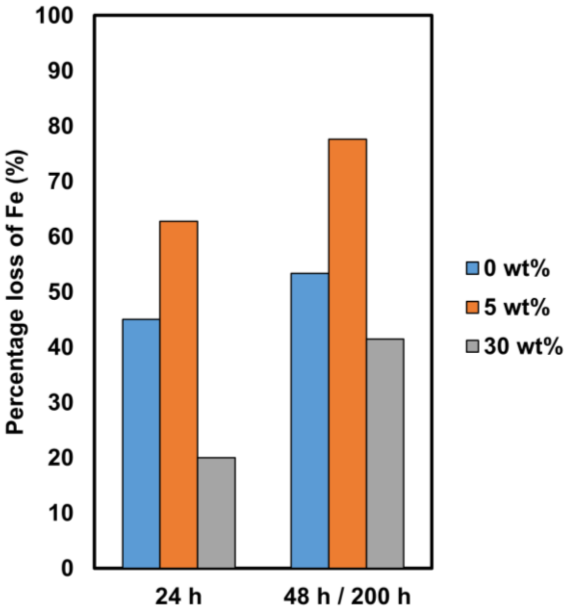

Figure S7. XRF results for tested anode catalyst layers with 0 wt%, 5 wt%, and 30 wt% Versogen ionomers. Durability tests were 48 h for 0 wt% and 200 h for 5 wt% and 30 wt%.

The total Fe loss was greatest for the 5 wt% sample. Compared to the 30 wt% sample, this can be attributed to decreased adhesion of the catalyst by the ionomer phase to the electrode surface. Compared to the 0 wt% ionomer sample, this likely occurred due to ionomer loss (evident in XPS analysis, **Fig. 7** and **Section S3**) leading to catalyst detachment for the 5 wt% sample, but not for the 0 wt% sample (which had no ionomer).

Tested membranes were measured for Fe content on the anode side to quantify catalyst transfer to the membrane from the transport layer. The results are summarized in **Table S2**.

Table S2. Fe loadings on tested membranes

Sample name	Loading (mg/cm^2)	Standard deviation (mg/cm^2)
0 wt% 24 h	0.018	0.002
0 wt% 48 h	0.014	0.002
5 wt% 24 h	0.041	0.007
5 wt% 200 h	0.014	0.004
30 wt% 24 h	0.033	0.003
30 wt% 200 h	0.057	0.004

Select tested electrodes were studied with XRD to look at changes to the crystal structure of the catalyst before and after testing. To quantify the relative changes to the $NiFe_2O_4$ and α-Fe_2O_3 phases,

characteristic peaks for each phase were identified based on the most prominent peak for each phase. For $NiFe_2O_4$, this was the peak at 35.7° and for α-Fe_2O_3 this was the peak at 33.2°. The ratio of the peak areas was determined and the change in this ratio was used to determine the relative loss of α-Fe_2O_3 compared to $NiFe_2O_4$.

S8. ICP-MS analysis of tested electrolytes

ICP-MS testing was conducted on collected electrolytes from the 30 wt% and 5 wt% 200 h durability tests. Aliquots were collected from the drain of the recirculating electrolyte reservoirs. Samples were not collected on all days, and all days collected are shown in **Figure S8**. The results demonstrate a selective loss of Fe versus Ni for the duration of the test. Fe content in the electrolyte did not increase with time though this does not necessarily mean that Fe loss was not continuously occurring. Dissolved Fe may have crashed out of solution, become embedded in the membrane, or plated elsewhere in the system, including at the cathode.

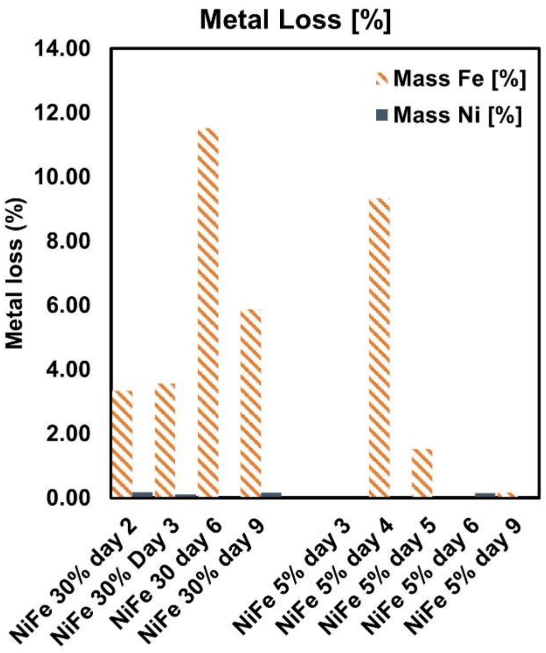

Figure S8. ICP-MS results for tested electrolytes from the 5 wt% and 30 wt% 200 h durability tests

S9. XPS analysis of tested electrodes

Ex-situ XPS data were collected for the C 1s, O 1s, F 1s, and N 1s regions for pristine and 200-h tested samples containing 5 wt% and 30 wt% Versogen ionomers. C 1s, O 1s, F 1s and S 2p spectra were collected for pristine and 200 h samples containing 10 wt% Nafion polymers.

The C 1s and O 1s spectra can also provide information on the state of N and F in the polymer; for example, the binding energy difference between C-F_2 and C-F_3 is much more pronounced in the C 1s spectrum. The C 1s spectra were deconvoluted into four peaks: C-C (defined at 284.8 eV), C-N/O (~286 eV), C=O (~288 eV), and C-F_2 (~292 eV).[12] The peak corresponding to C-F_3 would be expected at 293.5 – 294 eV, but was not observed in these measurements.[13] The peak at 286 eV cannot be definitively assigned to C-N or C-N^+,[14] so instead we will focus on the C-F_2 bond as an indicator of the polymer. While the peak intensity is quite low, it is clearly present in both pristine samples confirming the presence of the Versogen ionomer **(Figs. S9-10)**. As for the F 1s spectrum, the C-F_2 peak intensity declines after testing, indicating F loss from the polymer.

For the 5 wt% Versogen samples **(Fig. S9)**, before testing C-F and C-N bonds are apparent in the C1s spectra and O-F bonds in the O 1s spectra, indicating the presence of ionomer. After testing, there is a decrease in the C-N and C-F signals in the C 1s spectra. In the O 1s spectra, O-metal bonds dominate versus C-O signal, indicating a loss of ionomer relative to the catalyst. For both the pristine and 200 h tested samples, the F 1s and N 1s signals were low and no distinct features were observable.

For the 30 wt% Versogen samples **(Fig. S10)**, unlike the 5 wt% samples, the N 1s and F 1s signals are visible, reflective of a higher ionomer content in the catalyst layer. The N 1s signal aligns well with literature on the Versogen polymer; the peak at 402 eV corresponds to the ammonium group in the Versogen polymer.[13] In the C 1s spectra, there is again a relative loss of C-O leading to a spectra dominated by metal-O bonds after testing, indicating a greater loss of ionomer relative to catalyst. In the F 1s spectra, the signal matches that of organic F. The position of the C-F peak in the C 1s spectrum indicates that C-F_2 (rather than C-F_3) is present. Both N 1s and F 1s spectra show a loss in signal between the pristine and 200 h tested samples, further supporting that there is a loss of ionomer after testing.

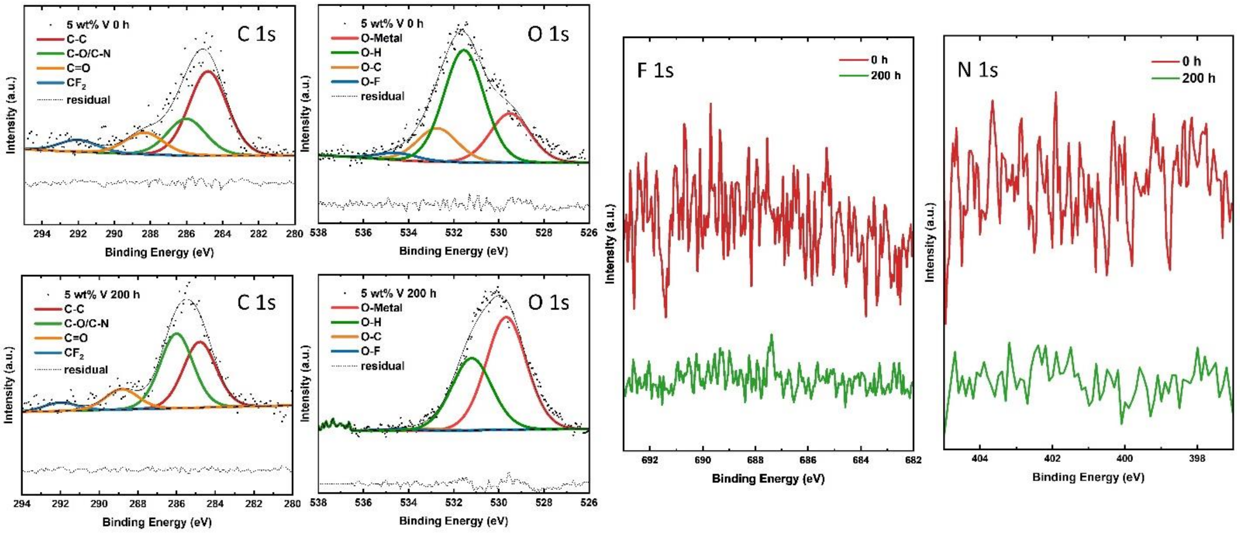

Figure S9. C1s, O 1s, F 1s, and N 1s results for XPS of pristine (top) and 200 h tested (bottom) electrodes with 5 wt% Versogen in the catalyst layer.

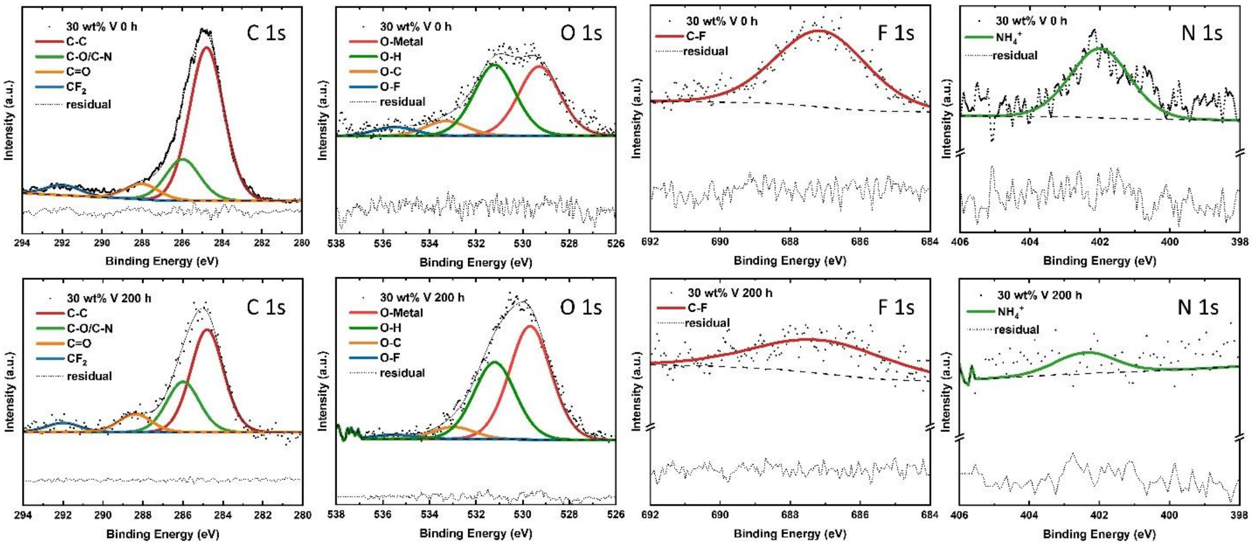

Figure S10. C1s, O 1s, F 1s, and N 1s results for XPS of pristine (top) and 200 h tested (bottom) electrodes with 30 wt% Versogen in the catalyst layer.

The 10 wt% Nafion samples **(Fig. S11)** show much higher F content than the Versogen samples. In the C 1s spectrum, the C-F_2 peak at 292.3 eV accounts for 67% of the C signal. The C-F_2 peak remains after the 200-h test, but the peak area is decreased to 30% of total C area. The pristine and tested samples show a large F 1s peak at ~687.5 eV, corresponding to the C-F_2. Prior to testing, the O 1s spectrum shows significant O-F character with a peak at ~535 eV, giving an O-F/O(H)-M ratio of 35%. In the post test sample, this decreases significantly to 3%, indicating loss of F. In addition, there is a small peak in the S 2p region, which corresponds to a doublet of metal-sulfate bonds at 169.5 and 170.7 eV. The S peaks are present after testing, but the signal was decreased, indicating that S was lost from the polymer.

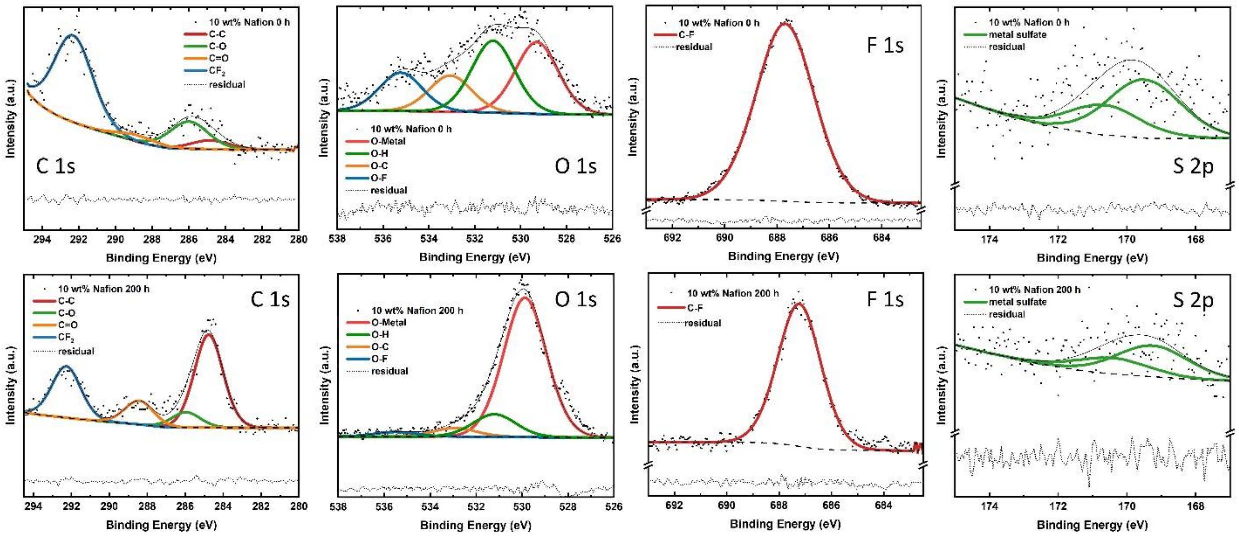

Figure S11. C1s, O 1s, F 1s, and S 2p results for XPS of pristine (top) and 200 h tested (bottom) electrodes with 10 wt% Nafion in the catalyst layer.

References

(1) Bard, A. J.; Faulkner, L. R. *Electrochemical Methods: Fundamentals and Applications*, 2nd ed.; Wiley, 2000.

(2) Fuller, T. F.; Harb, J. N. *Electrochemical Engineering*; John Wiley & Sons, 2018.

(3) González García, Á.; Nagelkerke, M. M. B.; Tuinier, R.; Vis, M. Polymer-Mediated Colloidal Stability: On the Transition between Adsorption and Depletion. *Advances in Colloid and Interface Science* **2020**, *275*, 102077. https://doi.org/10.1016/j.cis.2019.102077.

(4) Fischer, E. W. Elektronenmikroskopische Untersuchungen zur Stabilität von Suspensionen in makromolekularen Lösungen. *Kolloid-Zeitschrift* **1958**, *160* (2), 120–141. https://doi.org/10.1007/BF01503288.

(5) Wang, J.; Zhao, Y.; Setzler, B. P.; Rojas-Carbonell, S.; Ben Yehuda, C.; Amel, A.; Page, M.; Wang, L.; Hu, K.; Shi, L.; Gottesfeld, S.; Xu, B.; Yan, Y. Poly(Aryl Piperidinium) Membranes and Ionomers for Hydroxide Exchange Membrane Fuel Cells. *Nat Energy* **2019**, *4* (5), 392–398. https://doi.org/10.1038/s41560-019-0372-8.

(6) Mauritz, K. A.; Moore, R. B. State of Understanding of Nafion. *Chem. Rev.* **2004**, *104* (10), 4535–4586. https://doi.org/10.1021/cr0207123.

(7) Hoogeveen, N. G.; Cohen Stuart, M. A.; Fleer, G. J. Can Charged (Block Co)Polymers Act as Stabilisers and Flocculants of Oxides? *Colloids and Surfaces A: Physicochemical and Engineering Aspects* **1996**, *117* (1), 77–88. https://doi.org/10.1016/0927-7757(96)03699-0.

(8) Derjaguin, B.; Landau, L. Theory of the Stability of Strongly Charged Lyophobic Sols and of the Adhesion of Strongly Charged Particles in Solutions of Electrolytes. *Progress in Surface Science* **1993**, *43* (1), 30–59. https://doi.org/10.1016/0079-6816(93)90013-L.

(9) Verwey, E. J. W. Theory of the Stability of Lyophobic Colloids. *J. Phys. Chem.* **1947**, *51* (3), 631–636. https://doi.org/10.1021/j150453a001.

(10) Dixit, M. B.; Harkey, B. A.; Shen, F.; Hatzell, K. B. Catalyst Layer Ink Interactions That Affect Coatability. *J. Electrochem. Soc.* **2018**, *165* (5), F264. https://doi.org/10.1149/2.0191805jes.

(11) Shukla, S.; Bhattacharjee, S.; Weber, A. Z.; Secanell, M. Experimental and Theoretical Analysis of Ink Dispersion Stability for Polymer Electrolyte Fuel Cell Applications. *J. Electrochem. Soc.* **2017**, *164* (6), F600. https://doi.org/10.1149/2.0961706jes.

(12) National Institute of Standards and Technology. NIST X-Ray Photoelectron Spectroscopy Database, NIST Standard Reference Database Number 20,. **2000**, *20899*. https://dx.doi.org/10.18434/T4T88K.

(13) Dzara, M. J.; Artyushkova, K.; Foster, J.; Eskandari, H.; Chen, Y.; Mauger, S. A.; Atanassov, P.; Karan, K.; Pylypenko, S. X-Ray Photoelectron Spectroscopy Analysis of Nafion-Containing Samples: Pitfalls, Protocols, and Perceptions of Physicochemical Properties. *J. Phys. Chem. C* **2024**, *128* (20), 8467–8482. https://doi.org/10.1021/acs.jpcc.4c00872.

(14) Krivina, R. A.; Lindquist, G. A.; Yang, M. C.; Cook, A. K.; Hendon, C. H.; Motz, A. R.; Capuano, C.; Ayers, K. E.; Hutchison, J. E.; Boettcher, S. W. Three-Electrode Study of Electrochemical Ionomer Degradation Relevant to Anion-Exchange-Membrane Water Electrolyzers. *ACS Appl. Mater. Interfaces* **2022**, *14* (16), 18261–18274. https://doi.org/10.1021/acsami.1c22472.

(15) Xiao, J.; Oliveira, A. M.; Wang, L.; Zhao, Y.; Wang, T.; Wang, J.; Setzler, B. P.; Yan, Y. Water-Fed Hydroxide Exchange Membrane Electrolyzer Enabled by a Fluoride-Incorporated Nickel–Iron Oxyhydroxide Oxygen Evolution Electrode. *ACS Catal.* **2021**, *11* (1), 264–270. https://doi.org/10.1021/acscatal.0c04200.
